# Nitrothiophene carboxamides, a novel narrow spectrum antibacterial series: Mechanism of action and Efficacy

**DOI:** 10.1038/s41598-018-25407-7

**Published:** 2018-05-08

**Authors:** Shahul Hameed P, Nagakumar Bharatham, Nainesh Katagihallimath, Sreevalli Sharma, Radha Nandishaiah, Anirudh P. Shanbhag, Teby Thomas, Riya Narjari, Maitrayee Sarma, Purnendu Bhowmik, Prakruthi Amar, Rajani Ravishankar, Ramesh Jayaraman, Kubendran Muthan, Ramesh Subbiah, Vasanthi Ramachandran, V. Balasubramanian, Santanu Datta

**Affiliations:** 1 BUGWORKS Research India Pvt. Ltd., Centre for Cellular & Molecular Platforms, National Centre for Biological Sciences, GKVK, Bellary Rd, Bengaluru, Karnataka 560065 India; 2 Centre for Cellular and Molecular Platforms (C-CAMP), National Centre for Biological Sciences, GKVK, Bellary Rd, Bengaluru, Karnataka 560065 India; 3 St. John’s Research Institute, 100 Feet Rd, John Nagar, Koramangala, Bengaluru, Karnataka 560034 India; 4 TheraIndx Lifesciences Pvt. Ltd., Sy No. 27, Deganahalli, Budihal Post, Nelamangala, Karnataka 562123 India; 5 Syngene International Ltd., Plot 2 & 3, Bommasandra Industrial Estate - Phase-IV, Bommasandra-Jigani Link Road, Bengaluru, Karnataka 560099 India

## Abstract

The mechanism of efflux is a tour-de-force in the bacterial armoury that has thwarted the development of novel antibiotics. We report the discovery of a novel chemical series with potent antibacterial properties that was engineered to overcome efflux liability. Compounds liable to efflux specifically via the Resistance Nodulation and cell Division (RND) pump, AcrAB-TolC were chosen for a hit to lead progression. Using structure-based design, the compounds were optimised to lose their binding to the efflux pump, thereby making them potent on wild-type bacteria. We discovered these compounds to be pro-drugs that require activation in *E. coli* by specific bacterial nitroreductases NfsA and NfsB. Hit to lead chemistry led to the generation of compounds that were potent on wild-type and multi-drug resistant clinical isolates of *E. coli*, *Shigella spp*., and *Salmonella spp*. These compounds are bactericidal and efficacious in a mouse thigh infection model.

## Introduction

Although most bacterial strains possess over four thousand genes, only about four hundred of these are strictly essential for their survival. With the widespread rise in antibiotic resistance across various bacterial pathogens, a pivotal approach to address this major concern is to develop antibacterial compounds that specifically inhibit these essential but novel targets. Most often bacteria develop resistance either by target mutations that reduce the binding affinity of the drug or by “effluxing” these out of the cell; events that drastically reduce or abort the antibacterial effect of the compound. The efflux pumps of the RND family are significant contributors^1^ to the widespread multi-drug resistance (MDR) exhibited by Gram-negative bacteria. These pumps can efflux a wide range of compounds including structurally and chemically diverse antibiotics like fluoroquinolones (e.g., ciprofloxacin and levofloxacin), β-lactams (e.g., piperacillin, meropenem, and aztreonam), tetracyclines (minocyline), oxazolidinones (linezolid) and β-lactamase inhibitors (e.g., clavulanate and sulbactam)^2–4^. Owing to their wide-substrate specificity, overexpression of the RND efflux pumps often results in decreased susceptibility to antibacterial agents^1^. These observations are substantiated by improved MIC (minimum inhibitory concentration) to several antibiotics in efflux pump mutants or wild-type (WT) bacteria tested in the presence of efflux pump inhibitors^5,6^.

The dominant paradigm of antibiotic discovery efforts in the last three decades has been to identify unique small molecules that interfere with the functioning of an essential target while also having a concomitant adverse impact on the bacterial viability. Unfortunately such target-directed drug discovery efforts based on this “logical hypothesis” have met with limited success^7^. Although hits from enzyme based screens have been successfully optimized to leads with nanomolar potency against essential targets, these compounds strikingly lacked activity against WT bacteria^8,9^. In case of Gram-negative pathogens, this lack of MIC could be attributed to the efficient efflux of the inhibitors via the diverse set of efflux pumps present on the outer membrane. The latter observation is based on reports that potent inhibitors have MICs in pump deleted mutants like *E. coli* Δ*tolC*, but in contrast are totally ineffective against WT bacteria^8,9^. Target based exploration often resulted in hits that did not have whole cell activity. Alternatively, phenotypic screens started with hits that possessed anti-bacterial activity. However, the molecular target of such hits emerging from phenotypic screens were unknown. In the absence of target information, further optimization was often challenging and resulted in flat structure-activity relationship (SAR). Additionally, most WT active hits from commercial chemical libraries are compounds that lack drug-like properties (e.g., high logP), hit multiple targets, make lead identification and optimization a challenging process. Thus, even after investments of billions of dollars by large pharma companies, these approaches have failed to deliver new anti-bacterials^10^.

Since the elucidation of three-dimensional structures of the multi-subunit complex efflux pumps, most reports described strategies to inhibit pumps with small molecules as a strategy to inactivate them^11^. However, such approaches have a low probability of success either due to the presence of multiple families of pumps making the problem multifactorial or because the level of efficiency of these pumps vary depending on the stress or the milieu. Considering these hurdles, we explored a novel approach by designing antibacterial compounds that lose their interaction with the efflux pump and as a consequence escape efflux.

The primary RND efflux complex in *E. coli* consists of a pump protein (AcrB), a channel that traverses the outer membrane (TolC), and AcrA that connects TolC and AcrB. A proton-motive force drives the efflux pump complex^12^. The AcrAB-TolC efflux pump system and its homologs such as MexAB-OprM of *Pseudomonas aeruginosa* are well studied^2–4^. Initial three-dimensional structures of AcrB with various antibiotics reveal that AcrB is a homo-trimeric complex^12^ and antibiotics such as ciprofloxacin and nafcillin (PDB ID: 1T9U & 1T9W, respectively) interact either with the central or periplasmic cavity^13^. Recent reports of co-crystal structures of AcrB with small molecules reveal that it is assembled as an asymmetric trimer and it exists in three different conformations^14^. These are termed as Access/Loose, Binding/Tight and lastly the Extrusion/Open conformation respectively.

In the current study, we have devised a phenotypic-screen based cascade with a series of multiple evaluations using various efflux deficient *E. coli* strains. Compounds that were subjected to efflux exclusively via the AcrAB-TolC pump complex were identified and optimized to reduce binding to AcrB using structure-based design algorithm. This led to the identification of nitrothiophene carboxamide (NTC) based compounds that demonstrated potent activity against *E. coli*, *Klebsiella spp*., *Shigella spp*., and *Salmonella spp*. The NTC compounds are prodrugs that require activation by specific nitroreductases within the bacterial cell for their anti-bacterial activity. They are bactericidal *in vitro* and efficacious in a mouse thigh infection model.

## Results

### Hit identification and optimization

In the quest to discover novel hits against Gram-negative bacteria, a diverse set of 3000 compounds from a commercially available chemical library (Emolecules database) were screened against WT *E. coli* CGSC#7636 (BW25113), *E. coli* ∆*acrB* JW0451-2, and *E. coli* ∆*tolC* JW5503-1 in a whole-cell based growth inhibition assay. The reason for choosing specific knockout strains was to pick compounds with narrow differences in MIC values between the ∆*acrB* and ∆*tolC* strains and sieve out compounds possessing multiple efflux pump liabilities. Our objective was to progress those compounds that have specific AcrB efflux liability and can be optimized for WT antibacterial activity. Screening 3000 compounds yielded 181 active compounds (MIC ≤ 80 µg/ml) against *E. coli* Δ*tolC* and of these only seven of them had MIC against *E. coli* ∆*acrB* JW0451-2. None of these compounds had any activity against the WT. Seven hits that showed activity on Δ*acrB* were considered for further evaluation against 15 single gene knockouts of various efflux pump components (Table S1). MIC data indicated that all the 7 compounds were subjected to efflux only through AcrB (Table S1). Due to its potent MIC, and considering the typical hit evaluation parameters such as molecular weight (<500), lipophilicity (LogP/logD <5), number of hydrogen bond donors/acceptors (HBD < 5 and HBA < 10) and synthetic feasibility, **compound 7** was taken forward for further optimization.

**Figure 1:**
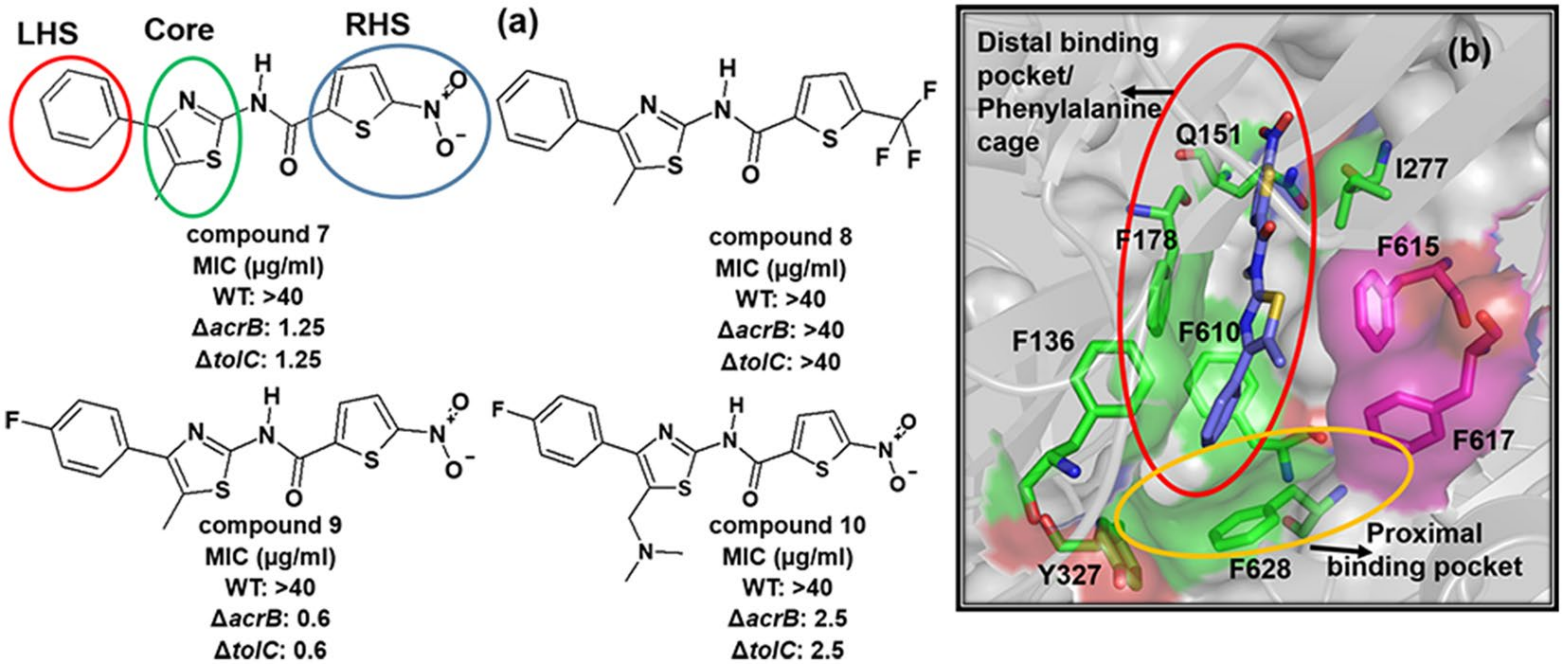
Initial rounds of hit optimization. (**a**) Chemical structures and associated MIC values on WT and efflux pump knockout strains. Important parts of the hit compounds are highlighted with circles and named accordingly. (**b**) Binding mode of compound 7 with AcrB efflux pump. Crucial residues of the hydrophobic pocket shown as sticks and important regions are highlighted with circles and named accordingly. The compound 7 depicted as blue sticks.

In order to decipher the SAR requirements for antibacterial activity, the middle methyl substituted thiazole core, and distal phenyl ring on the left-hand side (LHS) was kept constant (compound 8) and the initial chemistry efforts were directed towards changes on the right-hand side (RHS) nitro thiophene ring (Fig. 1a). None of these modifications yielded compounds with activity against WT bacteria. Instead, such changes made the compound weaker against ∆*acrB* and ∆*tolC* strains. These observations indicated that the RHS nitro thiophene group is essential for primary target interaction. In case of compound 9, an electronegative fluorine group at the para position of LHS phenyl ring retained the MIC against *acrB* and *tolC* mutant strains, whereas compound 10 with additional N,N′-dimethyl basic group on the thiazole ring (to compound 9) reduced MIC by four fold in ∆*tolC* compared to 9. However, none of these modifications provided any SAR handle towards mitigating efflux liability, implying that the nitro thiophene ring may be critical for interaction with unknown target/s that could be bringing about the growth inhibition of the efflux deficient strains.

Computational molecular docking simulations were performed to evaluate the interaction pattern of compound 7 with the AcrB efflux pump and for applying a structure-based design approach to reduce the efflux pump liabilities. The output (dock poses) of Autodock Vina^15^ blind docking in *Ec*AcrB protein structure was imported into PyMOL^16^ viewer and visualised. Similar computational docking studies of various antibiotics by Hiroshi Nikaido and group demonstrated that indeed most compounds bind to a phenylalanine rich aromatic cage^17^. We too observed that a high number of poses clustered in the distal binding pocket made primarily of aromatic residues such as F136, F178, Y327, F610, F615, F617 and F628. The RHS thiophene ring of compounds formed hydrophobic contacts with I277 residue and the nitro group of thiophene ring formed a hydrogen bond with Q151 residue (Fig. 1b). The middle core thiazole ring interacted with F178, and F615 residues and the LHS phenyl ring entered into the bottom of distal binding pocket which is lined by F136, Y327, F610 and F628 residues.

**Figure 2:**
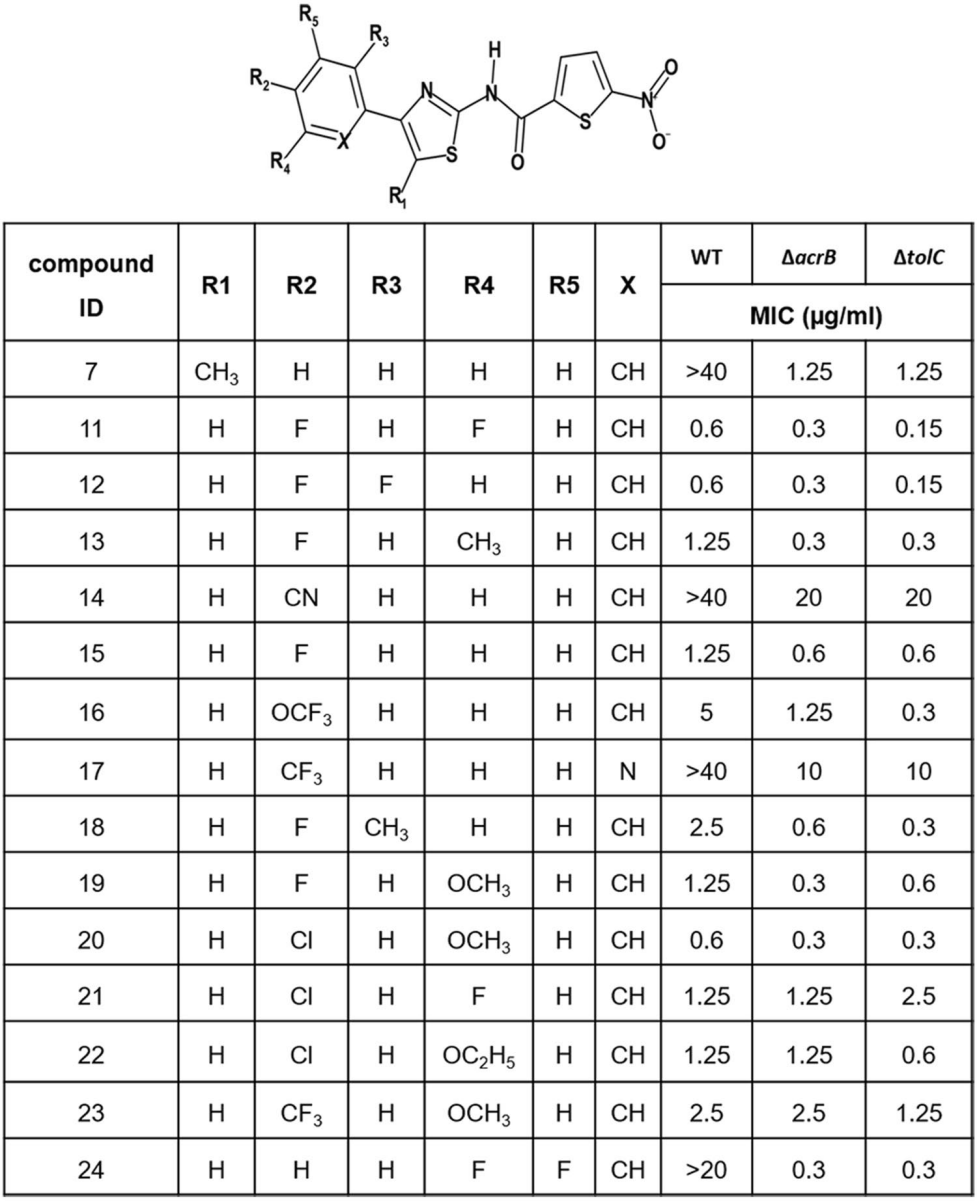
SAR of LHS ring with associated MICs on *E. coli* WT and pump knockouts.

**Figure 3:**
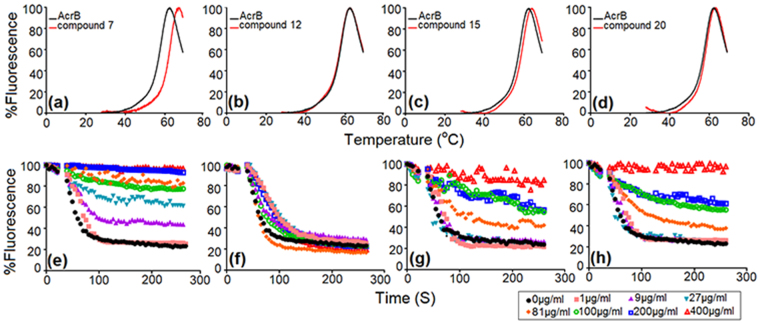
Evaluation of AcrB binding and efflux liability of NTC derivatives. Thermal shift assay (**a**–**d**), shows the melting curve of the AcrB apo-protein in black and with the compounds in red. A ∆Tm of  > 1**°**C implies binding and ∆Tm of <1 °C implies no binding; (**a**) ∆Tm = 4.13 °C for compound 7, (**b**) ∆Tm = 0.68 °C for compound 12, (**c**) ∆Tm = 1.75 °C for compound 15 and (**d**) ∆Tm = 1.86 °C for compound 20. Efflux assay (**e**–**h**), the curves depict time-dependent competition of Nile red with compounds in *E. coli* BW25113. Concentration of compound that is required for complete inhibition of Nile red efflux (i.e., T_efflux50_ >200 seconds); (**e**) 9 μM compound 7, (**f**) >400 μM compound 12, (**g**) 100 μM compound 15 and (**h**) 100 μM compound 20. A detailed table of T_efflux50_ at all tested concentrations of compounds is shown in supplementary Table S2.

Phenylalanine-Arginine Beta-Naphthylamide (PAβN) is a known efflux pump inhibitor that binds to the distal binding pocket/phenylalanine cage of AcrB^17^. Compound 7 that lacks MIC against WT *E. coli* (Fig. 2), exhibited potent activity (MIC < 0.3 µg/ml) in the presence of PAβN indicating that it binds to the phenylalanine cage before being subjected to efflux. Compound 7 binding to the distal site of AcrB binding pocket was further evaluated by its competition with Nile red in a whole-cell efflux assay. Earlier studies have proven that, besides the known inhibitors like PAβN, several tetracyclic compounds (doxorubicin, minocycline, chlortetracycline, doxycycline, and tetracycline) and tetraphenylphosphonium chloride interfere with the efflux of the dye Nile red^18^. In our experiments (Table S2), PAβN shows strong competition with Nile red by completely blocking its efflux at a low concentration (T_efflux50_ >200 Sec at 1 µM). Similarly, compound 7 slows down (Fig. 3 and Table S2) the Nile red efflux significantly (T_efflux50_ >200 Sec at 9 µM). Based on these observations we deduced that compound 7 binds to distal pocket/ phenylalanine cage of AcrB.

Next, we attempted to modify the LHS phenyl ring which was predicted to interact with the deep hydrophobic pocket that is lined with several aromatic/hydrophobic residues. Our primary intention with such an approach was to disturb the interaction with the phenylalanine cage residues. Subtle modifications capable of modulating electrostatic potential/electron density of LHS ring at ortho, meta and para positions of the phenyl ring were attempted (Fig. 2). Introduction of small electronegative fluorine at meta & para (compound 11) and ortho & para (compound 12) of LHS ring alleviated the efflux leading to WT activity (0.6 µg/ml). Additionally, an introduction of electron donating methyl and methoxy substituent at ortho or meta positions while keeping para fluoro substitution constant (compounds 13, 18 and 19) also provided similar activity against *E. coli* WT. Introduction of para nitrile group (compound 14) was detrimental as the compound showed >100 fold loss in MIC against ∆*tolC* compared to compound 12. This data demonstrated that para nitrile substitution might not be favourable for primary target interaction. Replacing para nitrile with fluoro (compound 15) brought back WT activity. The progression of compound 20 to 22 having para chloro substitution along with methoxy, fluoro and ethoxy groups on meta position of LHS ring respectively provided potent antibacterial activities in WT *E. coli*.

Thermal shift assay with CPM (7-diethylamino-3-(4′-maleimidylphenyl)-4-methylcoumarin) dye helped ascertain the differences in affinity of AcrB binding to compound 7 and compound 12. The ∆Tm is minimal in the case of compound 12 as compared to compound 7 (see Fig. 3a,b and Table S2). Compound 15 and compound 20 showed <2 **°**C shift in ∆Tm as compared to the protein alone (Fig. 3c,d). The compound 12 with a T_efflux50_ of 68 seconds is less competitive as compared to the initial hit compound 7 that has a T_efflux50_ of >200 seconds when tested with 400 µM of compound in the Nile red efflux assay (Fig. 3e,f). Dose response curves (7 doses from 1 µM to 400 µM) indicate that compound 15 and 20 compete with Nile red in the efflux assay while compound 12 does not show competition (Fig. 3f–h and Table S2). Both the efflux assay and the thermal shift analyses support that the compounds with wild-type MIC activity indeed have less efflux liability as compared to compound 7.

To discern the differences in interaction at the molecular level between compound 7 and compound 12, molecular dynamics (MD) simulations were performed for AcrB:ligand complexes. The reduced model (Figure S1a) of AcrB protein that was proposed by Vargiu & Nikaido and validated by several other groups^19,20^ were considered for the current study. Initial MD simulation trajectory analyses revealed that both the simulations converged (Figure S2a) within 3 Å backbone RMSD (root mean square deviation). Ligand RMSD analyses demonstrated an elevation of RMSD (around 10 ns simulation time) in case of compound 12 suggesting a probable difference in binding mode (Figure S2b). Further fragmentation of RMSD for RHS, LHS portions showed a drift in RMSD related to the LHS part and not from RHS (Figure S2c and d). To capture the binding mode differences, residual decomposition analyses (Table S3) was performed using 1500 snapshots (every 10^th^ snapshot from 10000 to 25000 ps). This analysis demonstrated that compound 7 indeed maintained all the hydrophobic interactions (Movie S1) with the phenylalanine cage as predicted by molecular docking (Figures S1b,c and S3a) whereas compound 12 lost several hydrophobic interactions and formed hydrophilic interactions with distal pocket residues (Figures S1d,e and S3b).

### Bactericidality and evaluation against clinical isolates

**Figure 4:**
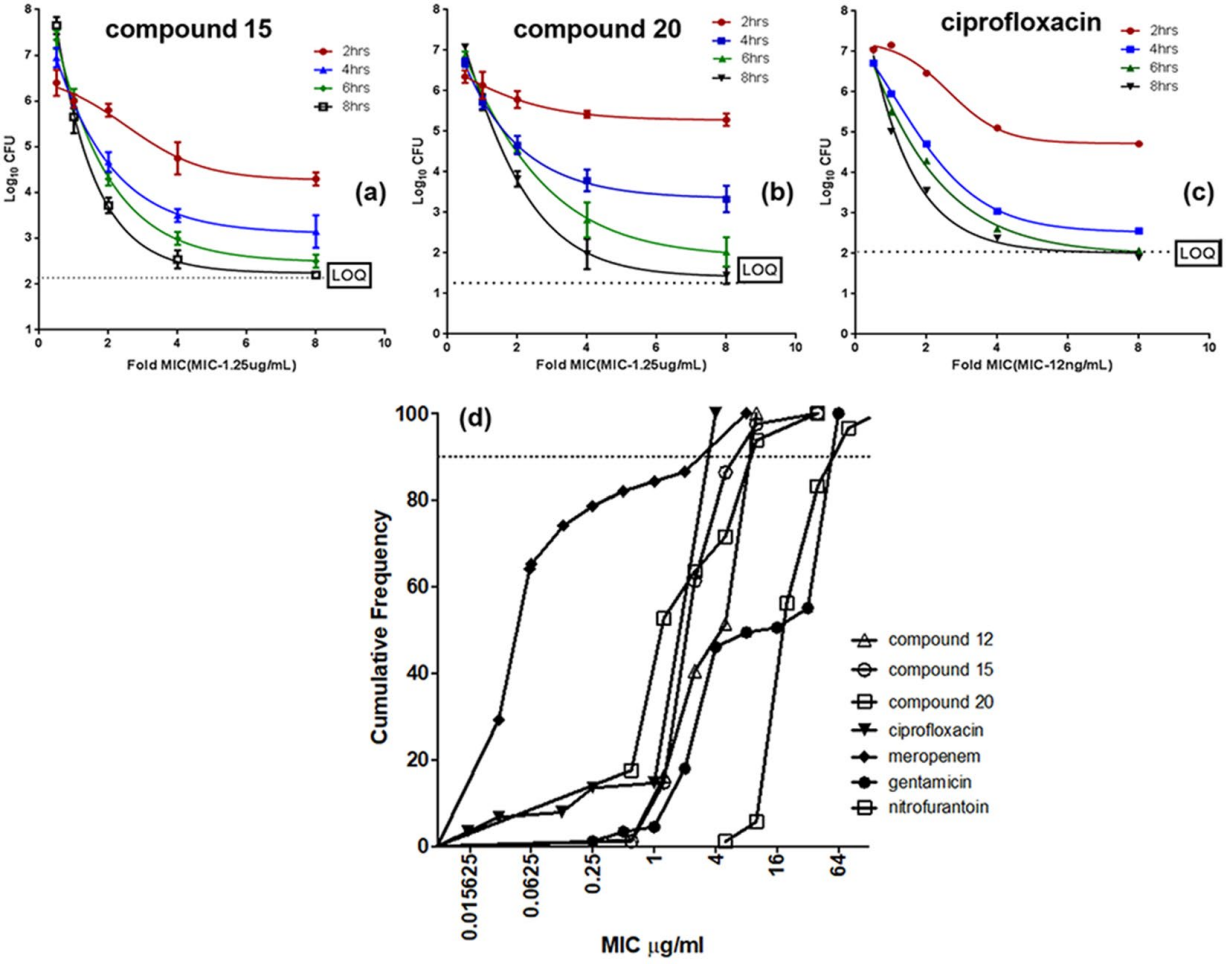
*In vitro* Time kill analysis of the compounds 15 (**a**), 20 (**b**) & ciprofloxacin (**c**) against *E. coli* BW24113. (**d**) MIC90 analysis of compounds 12, 15 & 20, along with standard drugs for a collection of recent uncharacterised clinical isolates of *E. coli* (St. John’s Medical Hospital, Bengaluru, India).

**C**ompounds 15 and 20 were evaluated for their bactericidal activities. Ciprofloxacin was used as a reference drug (Fig. 4a–c) for these assays. Killing kinetics data shown in Fig. 4 indicated that compounds 15 and 20 were as bactericidal as ciprofloxacin. The bactericidal activity of a compound can be assessed using two parameters. Firstly, the MBC/MIC ratio, wherein the minimum bactericidal concentration (MBC) indicates that concentration of the antibacterial agent that leads to 99.9% reduction in bacterial survival in a 24-hour period. The second parameter is the time taken to attain the 99.9% reduction. Compounds 15, 20 & ciprofloxacin achieved 99.9% reduction between 6–8 hours at 2–4X MIC. In addition, total kill (below the limit of quantitation, LOQ) was observed with both compounds at 8 hours and at 8X MIC. The kill rate appeared better for ciprofloxacin, reaching the limit of quantification at 6 hours and at 8X MIC.

Next, we evaluated compounds 12, 15 and 20 for their antibacterial activity against a panel of seventy five *E. coli* clinical isolates (Supporting File SI2). These clinical isolates were of recent origin and exhibited a range of susceptibilities to the standard drugs, ciprofloxacin, meropenem, nitrofurantoin and gentamicin. Four isolates were susceptible to the four standard drugs, and nine were resistant to all the four classes as per EUCAST breakpoint guidelines^21^. The remaining isolates were resistant to one or two of the drugs. The range of MICs, MIC50 (MIC of 50% of the tested isolates) and MIC90 (MIC of 90% of the isolates) are shown in Fig. 4d and Table S4.

### Cytotoxicity

Compounds 12, 15 and 20 were tested for cytotoxicity in A549 cell line using MTS ((3-(4,5-dimethylthiazol-2-yl)-5-(3-carboxymethoxyphenyl)-2-(4-sulfophenyl)-2H-tetrazolium)) assay. The IC_50_ were 16.9, 10.2, and 8.3 µg/ml, respectively. Staurosporine was used as a reference drug in this study (Figure S4).

### Target Identification

To identify the putative target, we performed a MIC screen with the compounds of interest against 700 *E. coli* mutants, each of them has a deletion of a single non-essential gene^22^. Those mutants that have an altered MIC could provide an insight of the potential target/pathway^23^ and eventually implicate the possible mode of action. The single gene knockout of *nfsB* showed a significantly elevated MIC (>40 µg/ml) as compared to the WT implying that this gene may be involved in the antibacterial activity of these compounds. Nitro-heterocyclic compounds are known substrates for nitroreductases NfsB and NfsA in *E. coli*^24–26^. Earlier studies have demonstrated that the biological activity of nitro-aromatic and nitro-heterocyclic compounds like nitrofurantoin is observed only after the reduction of the nitro moiety^25^. Since our current series of compounds are NTC derivatives, these were hypothesized to being activated by bacterial nitroreductases, in a manner similar to nitrofurantoin.

To test this hypothesis, we generated resistant mutants of the *E. coli* strain BW25113 for compound 11 and compound 15 by plating them on agar plates containing 2x, 4x, 8x and 16x MIC of the respective compounds. The resistance frequency was found to be 10^−7^ and 10^−8^ for compounds 11 and 15 respectively (Table S5). The *nfsB* gene and its regulatory regions in 10 mutants were sequenced and a frame-shift mutation was observed at base 347 (amino acid residue 116) in the presence of both compounds (compound 11 and 15). The identified mutations were mapped to its 3D structure (PDB ID: 1YKI) to understand the functional relevance of such variations. As shown in Figure S5a, residues after 116 were crucial for FMN (Flavin mononucleotide) tricyclic ring and phosphate binding. It is presumed that the frameshift mutation disturbs the active site pocket thereby altering the enzyme activity (Figure S5a). A similar effect was expected from second frameshift mutation, which appeared at base 423 (amino acid residue 141). A point mutation (V143F) was observed in case of compound 15. The V143 residue was within 4 Å vicinity to FMN binding pocket, and the side chain of the valine was surrounded by several hydrophobic residues (Figure S5b). Valine is a small hydrophobic residue and mutations with bulkier aromatic side chain (phenylalanine) could cause severe steric clashes with surrounding residues thereby disturbing the architecture of the FMN binding pocket. Mutations were not observed in the *acrB* gene of these mutants, confirming that the resistance was not due to changes in the *acrB* that could have caused increased efflux of the compounds.

To assess the biological relevance of the interacting protein and establish the link between nitroreductases to the antibacterial activity of NTC compounds, single (Δ*nfsA* or Δ*nfsB*) and double knockouts (Δ*nfsA* & Δ*nfsB*) of nitroreductases were generated by phage transduction using strains from the Keio collection^22^. The rationale behind the selection of these knockouts was based on the observation that the drug nitrofurantoin showed an elevation of the MIC in single knockouts and about 8-fold elevation in the double knockout, implicating the role of both enzymes in nitro group reduction. Similarly, compounds 12, 15 and 20 showed partial MIC elevation in single knockouts (Δ*nfsA*) and were completely inactive in Δ*nfsA* and *ΔnfsB* double knockout strain (Table S6). The compound 14 (negative control) was inactive and did not show any difference in either single or double knockout strains.

### Nitroreductase assay

**Figure 5:**
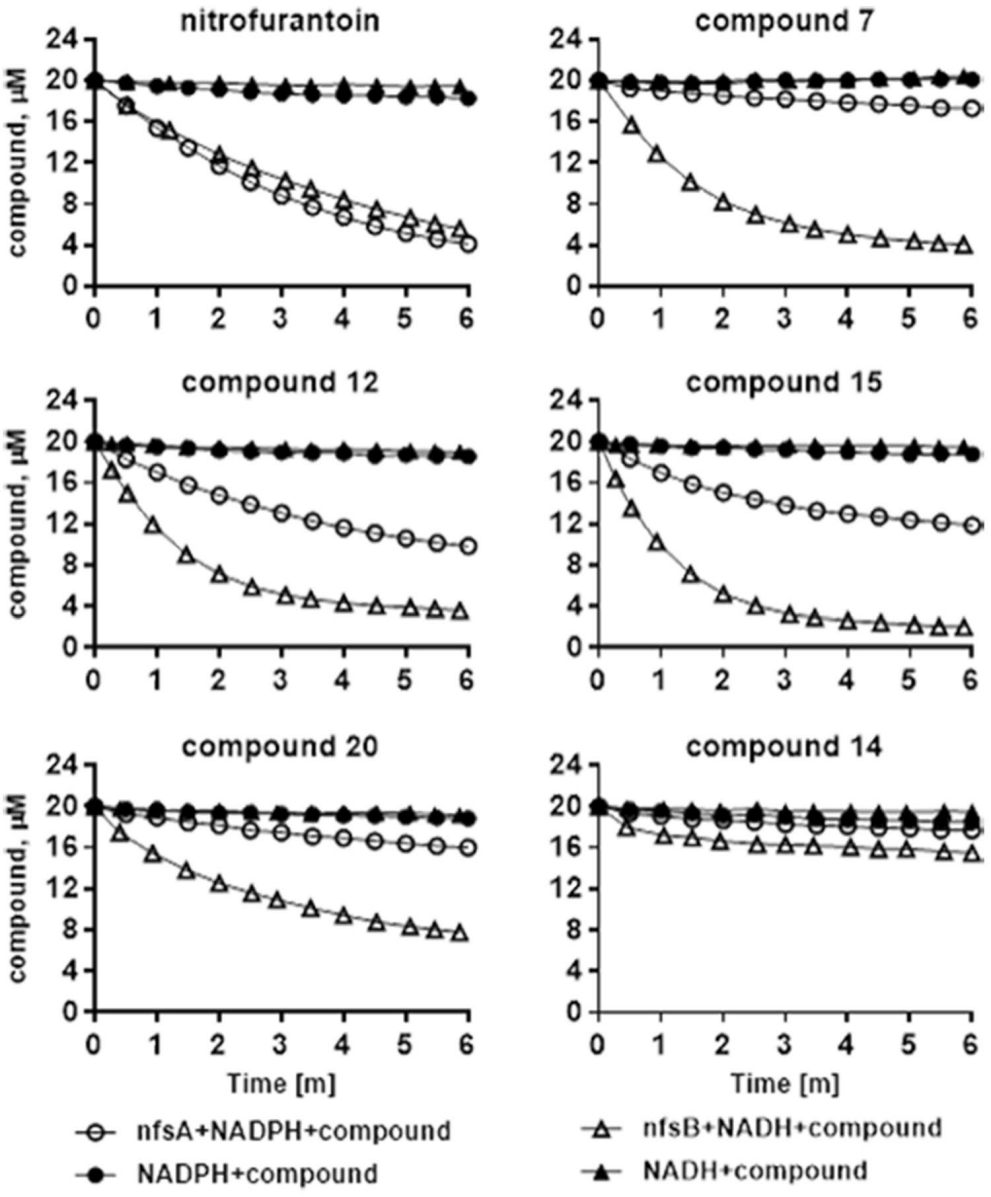
NfsB and NfsA nitroreductase enzyme activity, the reactions are initiated with 40 µM of co-factor NADPH and NADH respectively. The depletion of the co-factor is monitored in a fluorescence assay that is used to measure the reduction of the compounds 7, 12, 15, 20, and 14 along with a positive control nitrofurantoin over a period of 6 minutes.

To establish the biochemical activity and also validate the microbiological results that showed better conversion by Δ*nfsA* than Δ*nfsB* strain (Table S6), the two enzymes were purified (Figure S6) and their activities were assessed with the NTC compounds. It was seen that the representative set of compounds having WT MICs are more efficiently converted by NfsB into their active forms compared to NfsA as shown in the activity profile of individual compounds with both NfsA and NfsB nitroreductases using NADPH and NADH as co-substrates respectively (Fig. 5). Correlation of enzyme activity with that of ∆*nfsA* MICs suggests that the cellular activity of NfsB helps in retention of WT MIC by compounds even in the absence of NfsA. Compound 14 has no WT MIC and is not converted by either NfsA or NfsB in the biochemical assay providing evidence for their role in the activation of these compounds into their antibacterial forms within the cells. The low solubility of the compounds/substrates under the NfsA and NfsB assay conditions precluded determination of their K_m_. Therefore, a quantitative measure of the test compound affinities towards the enzyme was obtained by comparing the slopes of the initial velocities of the reactions at a concentration of 40 μM NADH/NADPH and 10 nM NfsA or 70 nM NfsB with 20 μM compounds (Table S7).

### Pharmacokinetics and *In vivo* Efficacy

These studies were performed as per CPCSEA, Govt. of India approved protocols (Registration number 1610/RO/c/12/CPCSEA), and also following approval by the Institutional Animals Ethics Committee (IAEC). The pharmacokinetics (PK) of compounds 12 and 15 was performed in BALB/c mice following either as a single intravenous dose of 2 mg/kg or single oral dose of 10 mg/kg. All the mice were visibly normal post dose and throughout the study. The Non-compartmental pharmacokinetic analysis for compounds 12 & 15 is shown in Tables S8 and S9. These compounds exhibited moderate to high clearance, with an oral bioavailability of approximately 15–25%. Following 100 mg/kg oral dose, exposures achieved were sufficiently above the MIC, which prompted the evaluation of the compounds in an *in vivo* efficacy study.

**Figure 6:**
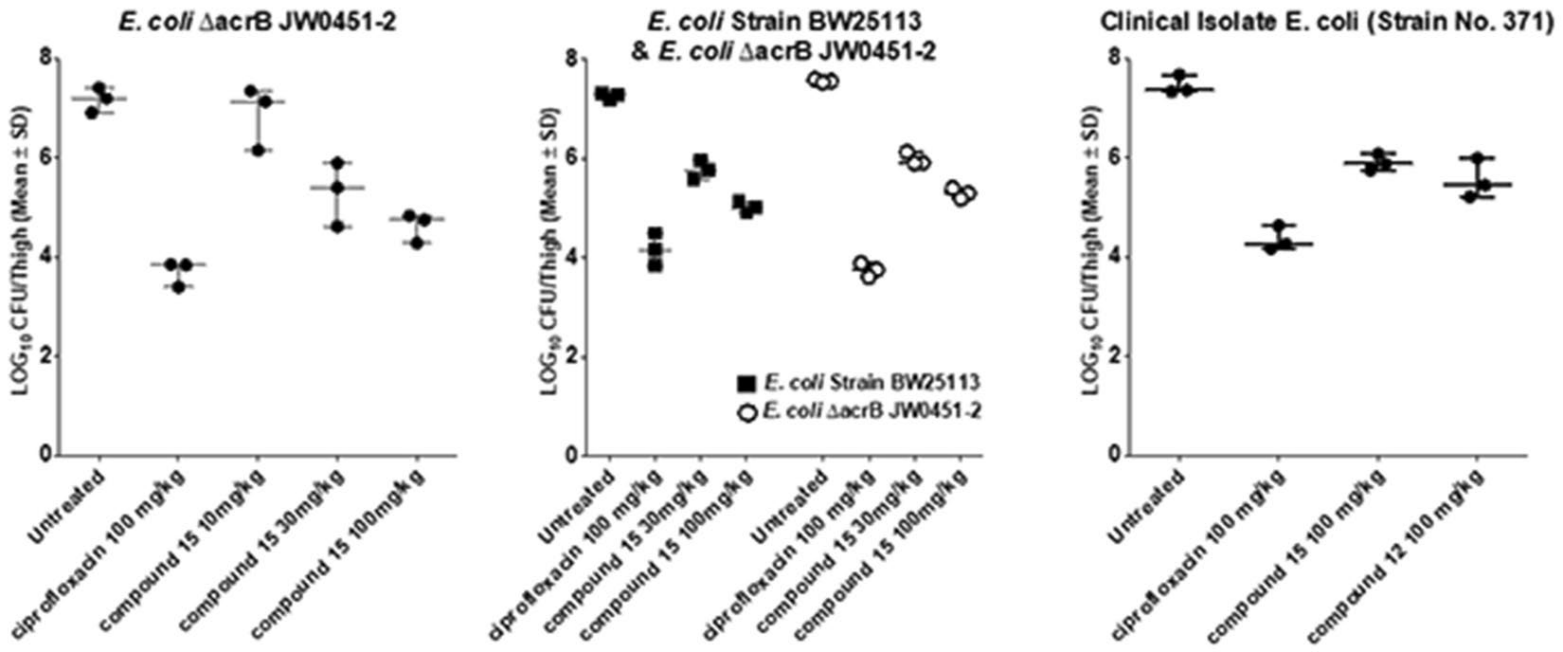
Animal efficacy evaluation. Bacterial load from infected thighs following per oral treatment with Ciprofloxacin 100 mg/kg or compound 15; 10, 30 or 100 mg/kg vs. *E. coli* ∆*acrB* JW0451-2; ciprofloxacin 100 mg/kg or compound 15; 30 or 100 mg/kg vs. *E. coli* ∆*acrB* JW0451-2 or *E. coli* BW25113; ciprofloxacin, compound 12 or compound 15 at 100 mg/kg tested against a recent *E. coli* clinical isolate strain No. 371, obtained from St. John’s Medical Hospital, Bengaluru.

In a thigh infection BALB/c mice model, compound 15 was found to be significantly bactericidal with a clear dose response when tested against an efflux pump deficient *E. coli* strain, ∆*acrB* JW0451-2, the WT *E. coli* strain BW25113 and a clinical isolate of *E. coli* (Fig. 6a–c and Table S10). No difference was observed between the efficacy of the efflux deficient and competent WT strain, indicating that the absence of any efflux liability under *in vivo* conditions. Compound 12 was tested only against the clinical isolate and was found to be significantly bactericidal (Fig. 6c and Table S10).

## Discussion

AcrAB-TolC and its RND homologs in Gram-negative pathogens are involved in the efflux of a wide range of antibiotics and hence considered as the most clinically relevant pumps. AcrB inactivation either by deletion or with inhibitors, triggers overexpression of other efflux pumps such as AcrF or AcrD that could aid in making the bacteria refractory to antibiotic action^27–29^. On the other hand, the efflux mitigation strategy described here resulted in neither compensatory activation of other pumps nor any mutations in AcrB. These conclusions are based on the observation that there is no difference in MIC between Δ*acrB* & Δ*tolC* knockouts and absence of any changes in *acrB* gene sequence in the mutants raised against these compounds. Our MD simulations approach demonstrated that the interactions with conventional hydrophobic cage residues diminished for compound 12 while compound 7 that has efflux liability maintained the interactions (Figures S1c,e and S7a,b). Compound 12 showed variable interaction pattern by forming hydrogen bond interactions with G288 and S287 (Figures S1e and S3b). An earlier report indicated that G288 is one of the critical hotspot residues in the distal binding pocket and G288D mutation in MDR clinical isolates of *Salmonella typhimurium* primarily altered specificity towards antibacterial drugs^20^. To validate the MD simulation observations, we also modelled compound 24 and it revealed an interaction pattern similar to the efflux liable compound 7. It did not show hydrophilic interactions with G288 or S287 but maintained all hydrophobic interactions (Figure S3c). These observations were confirmed by evaluating MICs against *E. coli* WT and pump deletion mutants, which demonstrated compound 24 to be efflux liable (Fig. 2). SAR studies of the compounds 11, 12, 15 and 24 revealed that the p-fluoro group is vital for overcoming efflux. Among these four, only compound 24 lacks the p-fluoro group, hence, behaves like compound 7. Small hydrophobic substitutions such as methyl or methoxy at the para position also help to overcome efflux. Additionally, SAR studies revealed that the modification of unsubstituted LHS phenyl ring altered the interaction pattern with the phenylalanine cage along with hydrophilic interactions with the hotspot residues, and thus explaining the modulation of efflux. These observations were further corroborated by Thermal shift based binding assay and the dose-dependent Nile red competition assay.

Some of the WT active hit compounds exhibited bactericidal activities comparable with known antibacterial drugs along with potent MIC90 on multi-drug resistant *E. coli* clinical isolates. Several pathogenic *E. coli* strains were resistant to four different classes of known antibiotics whereas the NTC derivatives are active on most of them (Table S4). Mode of action studies led to the identification of NfsB as the enzyme that activates these compounds that brings about the antibacterial effect. Single and double gene knockouts of *E. coli* nitroreductases, the NfsA & NfsB enzyme assays further substantiated that NfsB is the primary activator. Compounds within the series showed moderate MIC elevation in single gene knockout (Δ*nfsA* or Δ*nfsB*) strains and were completely inactive in the double gene knockout (Δ*nfsA* & Δ*nfsB*) strain. This evidence demonstrated the specificity of NTC activation by NfsB and NfsA unlike nitrofurantoin that is partially active even in the double knockout strain implying its activation by other non-specific enzymes (Table S6). Co-crystal structure of *E. coli* NfsB bound to cofactor FMN and nitrofurazone revealed that NfsB was a dimeric protein of 217 amino acids per polypeptide chain with one FMN cofactor per subunit^30^ and two nitrofurazone compounds were observed near FMN binding pocket (PDB ID: 1YKI). Molecular docking predicted that the binding mode for NTC derivatives with NfsB was such that the nitro group occupied an identical position to that of nitrofurazone warhead. The remaining part of NTC derivative bridges the dimer interfaces of NfsB (Figure S8a,b). Upon comparing *E. coli* NfsB sequence with its homologs from other pathogenic bacteria like *Klebsiella pneumoniae*, *Shigella flexneri*, *Shigella sonnei* and *Salmonella typhimurium*, a high sequence identity of ≥80%, as well as active-site pocket similarity was observed (Figure S8c). This analysis implies that these NTC derivatives could possess potent antibacterial activities against these bacterial pathogens too. *K. pneumoniae* nitroreductase *nfnB* on a plasmid vector was used to genetically complement the *E. coli* triple knockout of *nfsA, nfsB* & *acrB* and test the MICs of the NTC derivatives against this strain. The NTC compounds when tested against such a complemented strain, revealed that the heterologous nitroreductase from *K. pneumoniae* is able to activate them resulting in the MIC being restored in the triple knockout *E. coli* strain (Table S11). Evaluation of compounds 12, 15 and 20 against WT *K. pneumoniae* ATCC strain revealed reasonable MICs. The 4-8 fold lower potency of these compounds in comparison to *E. coli* WT MIC could be attributed to differences in either compound permeability and/or efflux pumps in *K. pneumoniae*. Nevertheless, this data demonstrated that the *E. coli* AcrB efflux mitigation strategy indeed holds good with the *K. pneumoniae* AcrB homolog. Similarly, potent MICs were also observed against clinical isolates like *Shigella flexneri*, *Shigella sonnei* and *Salmonella typhimurium* (Table S11).

Our work describes an innovative strategy to discover novel antibacterial compounds. The approach involves overcoming efflux liability by reducing the binding to AcrB, thereby mitigating their efflux liability. We started with efflux liable compounds and optimised them to obtain activity against wild-type bacteria. Such a strategy paves the way for the identification of novel antibacterial chemotypes that are pivotal to tackle the resistance risk posed by multi-drug resistant bacterial strains.

## Methods

### Determination of MIC and MBC

MIC (Minimum Inhibitory Concentration) of wild-type *E. coli* CGSC#7636 (BW25113), *E. coli* ∆*acrB* JW0451-2, and *E. coli* ∆*tolC* JW5503-1 were determined  in M9 medium according to Clinical Laboratory Standards Institute (CLSI) guidelines. The inoculum used for all the experiments is derived from a single seed lot that is maintained as glycerol stocks at −80 °C and are sub cultured on LB plate for isolated colonies. Single colony of each strain is grown in M9 medium. All test compound stocks and dilutions are prepared in DMSO. Serial two-fold dilutions of compounds are prepared separately, with the concentrations ranging from 2 mg/mL to 0.015 mg/mL. To 150 µl (3–7 × 10^5^ CFU/ml) of bacterial culture in 96 well microtitre plates, 3 μL compound from each of the dilutions is added to respective wells to obtain final concentrations ranging from 40 µg/mL to 0.3 µg/mL of the test compounds. Media control, culture control and appropriate reference drug controls are included. The plates are packed in gas permeable polythene bags and incubated at 37 °C overnight. Growth is monitored by checking absorbance at 600 nm (A_600_). Minimum inhibitory concentration (MIC) is taken as the concentration that result in a growth inhibition of ≥80%. The samples from MIC and above concentrations are plated for MBC (Minimum Bactericidal Concentration). The samples are diluted 1:10 and plated on LB agar plates. Plates are incubated at 37 °C overnight and number of surviving colonies are enumerated. MBC is considered as the lowest concentration which shows 99.9% kill from the start CFU. The killing kinetics is determined by enumerating survivors at various time points following compound exposure. *E. coli* cells (~10^7^ CFU/ml; A_600_ of 0.1) are treated with various concentrations (40 µg/mL to 0.3 µg/mL) of compounds in a 96-well microtitre plate. At various time intervals (0 to 8 h), 30 μl volumes of treated cultures are serially diluted and plated on LB agar plates. The plates are incubated at 37 °C overnight, and the bacterial colonies are enumerated. Data is expressed as the log_10_ CFU for each drug treatment.

### Resistant mutations prediction

Spontaneous resistant mutants are raised against compounds using a single step selection method. Mid-logarithmic phase culture of *E. coli* is centrifuged and concentrated 10-fold to achieve a bacterial number of ~10^9^ CFU/mL. Varying dilutions of the bacterial culture are plated onto compound containing plates (corresponding to 2x, 4x, and 8x MIC concentration). Appropriate dilutions of the bacterial culture are also plated on drug-free LB agar to enumerate the bacterial numbers in the culture. Plates are incubated for 24 hrs at 37 °C and the CFUs in drug containing and drug free plates are enumerated. The spontaneous rate of resistance was calculated by dividing the number of colonies on drug containing plates (at a given concentration) divided by the total number of viable bacteria estimated on drug-free plates. Resistant colonies are randomly picked from the drug containing plates and grown in MH broth to determine their level of resistance against the specific, compound as well as, other standard drugs with different mechanisms of action.

### Target Identification

The wild-type *E. coli* strain BW25113 and the entire Keio collection of knockouts^22^ is maintained as glycerol stocks at −80 °C in 96-well deep well blocks. Using a proprietary algorithm, we have identified about 700 non-essential gene knockouts that can be used for target identification. These are sub cultured in 150 µl of LB broth (Kanamycin 50 ug/ml, except for the wild-type strain) in 96 well microtiter plates and then adapted into M9 medium with 0.4% glucose. From these plates, a 2 µl inoculum of 10^6^ to 10^7^ CFU/ml is transferred to 150 µl per well of medium in microtitre plates containing compound 12 or compound 20 at 0, 0.6, 0.8 and 1.25 µg/ml. Plates are incubated overnight at 37 °C and growth or its inhibition was monitored by measuring the absorption (A_600_) using a plate reader. Cultures of single gene knockouts that show an altered MIC as compared to that of the wild type are scored as ‘Hits’.

#### Over-expression and purification of AcrB

AcrB overexpression protocol is adopted from Du. *et al*.^31^. Full length *acrB* gene was amplified from *E. coli*. BW25113 using Forward Primer 5′-GCCCATATGCCTAATTTCTTTATCGA-3′ Reverse Primer 5′-GCCGCTATTCAATGATGATCGACAGTAT-3′ and cloned into the plasmid pET21a. The resultant plasmid that would express AcrB with a C-terminal His-tag is transformed into *E. coli* strain C43(DE3). The cells are induced on reaching A_600_ = 0.6 and transferred to 18 °C. The culture is harvested by centrifugation and re-suspended in Buffer A (100 mM Sodium Phosphate, 100 mM NaCl, 10 mM Imidazole, pH = 7.4, 100 µg/ml protease inhibitor cocktail from Roche and 5 U/ml DNase I and 5 mg/ml lysozyme). The cells are lysed at 15000 psi using Emulsiflex and centrifuged at 8000 g for removing cell debris. The membrane is isolated by centrifuging the resultant supernatant at 125000 g (see Coomassie stained SDS-PAGE of the purified protein Figure S9). Membrane solubilisation is done using Buffer A and 1.5% DDM (n-Dodecyl-β-D-Maltoside). Ni-NTA chromatography is used to purify the His-tagged protein and eluted in Buffer A with 300 mM Imidazole and 1.5% DDM.

### Thermal Shift Assay

The assay is performed according to protocols established elsewhere^32^. CPM (N- [4-(7-diethylamino-4-methyl-3-coumarinyl) phenyl] maleimide) is a thiol specific fluorochrome (Excitation 387 nm, Emission 463 nm) and is non-fluorescent in an unbound state. The proteins, on thermal unfolding exposes cysteines allowing the CPM to bind and fluoresce. Here, AcrB protein at a final concentration of 0.125 µM along with 20 µM of CPM in 50 mM HEPES (pH 7.5), 300 mM NaCl, 5% glycerol and 0.03% DDM is used for the assay. The thermal denaturation is performed in a fluorimeter that is heated from 25 to 90 °C at a ramp rate of 1 °C/min. The Tm is calculated by analysing the melting profiles using Boltzmann Sigmoid function (GraphPad Prism) and ∆Tm is calculated by measuring the difference between the Tm of inhibitor bound protein and the apo-protein.

### Nile red efflux assay

The Nile red assay is adopted from Bohnert *et al*.^18^. An overnight culture of *E. coli* strain BW25113 is harvested and is resuspended in 20 mM potassium phosphate buffer (pH 7.0) containing 1 mM MgCl_2_ (PPB). The cells are adjusted to an A_660_ of 1.0 in PPB and aliquoted into glass tubes (2 ml each). Carbonylcyanide-m-chlorophenylhydrazone (CCCP; a protonophore that uncouples the electron transport chain and thus inhibits AcrB mediated efflux) is added to a final concentration of 10 µM (5 mM stock solution in 50% DMSO) and incubated for 15 minutes before adding the potential competitor at desired concentrations. Nile red is then added (stock solution of 5 mM in 10% dimethyl formamide-90% ethanol [vol/vol]) to a final concentration of 5 µM. The cell suspension is incubated at room temperature on a shaker for 3 hours. The cells are harvested and resuspended in 2 ml PPB in the presence of the competitor. The cell suspension is aliquoted in a fluorimeter cuvette, to which, 100 µl of 1 M Glucose is added to activate the electron transport chain. The rate of efflux is measured by T_efflux50_, it is the time taken for 50% of Nile red to be effluxed out in presence of the compounds. The fluorescence is measured (Excitation 552 nm, Emission 636 nm) for 200 seconds.

### Cloning, expression and purification of *E. coli* nfsA and nfsB

The *nfsA* gene was PCR amplified using the primers 5′TTCCATGGATATCATTTCTGTCGC3′ and 5′CTTCCATGGATATCATTTCTGTCGC3′. Likewise, the *nfsB* gene was amplified using the primers 5′TTCCATGGATATCATTTCTGTCGC3′ and 5′CTTCCATGGATATCATTTCTGTCGC3′ from *E. coli* BW25113 and cloned into the pET28a vector to produce pET-*nfsA* and pET-*nfsB* plasmids. Recombinant NfsA and NfsB proteins are over-expressed using these plasmids in *E. coli* BL21(DE3) strain following standard procedures^33^. In brief, the pET-*nfsA* and pET-*nfsB* plasmid transformed BL21(DE3) cells are grown in 300 ml LB-broth with 50 μg/ml Kanamycin at 37 °C to an A_600_ of 0.6, induced with 200 μM IPTG and continued incubation for 3 hours before harvesting the cells. The soluble His-tagged NfsA and NfsB proteins are purified by Ni-NTA affinity chromatography followed by Gel permeation chromatography elution using 25 mM Tris-HCl, pH 8 containing 150 mM NaCl. The fractions corresponding to dimer peaks are pooled, concentrated (see Coomassie stained SDS-PAGE of the purified protein Figure S6), and stored at −70 °C after addition of 10% glycerol and 1 mM EDTA. Protein concentrations are determined using BSA as a standard with Bradford reagent.

### Nitroreductase activity assay

Enzyme reducing each of the substrates are measured by monitoring the concomitant oxidation of NADPH by NfsA and NADH by NfsB in a fluorescence based assay. The reduction in fluorescence due to the NADH oxidation (excitation 350 nm, emission 460 nm) and NADPH oxidation (excitation 340 nm, emission 485 nm) is recorded using a TECAN Infinite M200Pro spectrophotometer, in half area solid black microtiter plates. This change in fluorescence directly corelates to the reduction of the nitro-compounds in the reaction. The NfsA assays is performed by initiating the reactions with 40 μM NADPH and 10 nM NfsA in 50 mM Tris-HCl (pH 7.4), 5 mM EDTA against 20 μM substrate (5 μl of compounds/substrate dissolved in 100% DMSO) in a total reaction volume of 100 μl at 25 °C. Similarly, the NfsB assay is also performed by initiating the reactions with 40 μM NADH and 70 nM NfsB in 50 mM Tris-HCl (pH 7.4), 5 mM EDTA against 20 μM substrate in a total reaction volume of 100 μl. These are the optimal conditions arrived at by titrating the enzyme between 2.5 nM–100 nM, NADH/NADPH between 10 and 100 µM, DMSO between 0 and 15% for its tolerance and solubility of the compounds/substrate. All reactions are performed in replicates of two or more and the fluorescence measurements are made at 10 second intervals for 5–10 minutes.

### Measurement of reaction rates for ranking of compounds

Low solubility of the compounds/substrates under the NfsA and NfsB assay conditions precluded determination of their K_m_. To establish a quantitative measure of the test compound affinities towards the enzyme, an approach of comparing the slopes of the initial velocities of the reactions at a fixed concentration of 40 μM NADH/NADPH and 10 nM NfsA or 70 nM NfsB with 20 μM compounds was adopted. The relative fluorescence units (RFU) obtained due to NADPH (excitation 340 nm, emission 485 nm) or NADH (excitation 350 nm, emission 460 nm) oxidation of the first 90 sec reaction is used to compute the slopes using linear regression analysis on GraphPad Prism. Appropriate blank reactions are included to account for background fluorescence/quenching from each of the components such as DMSO, the compound itself and auto-oxidation of NADPH or NADH. Activity of NfsB with a known substrate such as nitrofurantoin is used as a benchmark to plot the relative activities with all the test compounds. Though this data does not provide a precise measure such as k_cat_ and K_m_, comparison of the slopes of these initial velocities enables ranking of the compounds based on their affinity towards the enzyme.

#### Cytotoxicity studies

Cytotoxicity of the compounds was evaluated using MTS ((3-(4,5-dimethylthiazol-2-yl)-5-(3-carboxymethoxyphenyl)-2-(4-sulfophenyl)-2H-tetrazolium)) assay. A-549 cells were seeded in a 96 well plate at a density if 1.5 × 10^4^ cells/well (100 μL). The cells were cultured in a CO_2_ incubator at 37 °C. A ten point concentration curve was set up starting from 5 nM to 100 μM with 3 fold dilution along with positive, negative and blank controls. The final concentration of DMSO was maintained at 1% throughout the plate. The compound treatment was carried out for 24 hrs. After incubation MTS reagent was added followed by 30 min incubation. The absorbance was measured at 490 nm. Data analysis was done using GraphPad Prism 6.0.

### Autodock molecular docking

Blind docking experiments were performed for the hit molecule (compound 7) by Autodock Vina 1.1^15^. The minocycline bound *Ec*AcrB crystal structure (PDB ID: 4DX5) was considered for these experiments. AutoDock Vina 1.1, is an open-source program for docking simulations. It uses the Iterated Local Search global optimizer algorithm in which a succession of steps consisting of a mutation and a local optimization are taken, with each step being accepted according to the Metropolis criterion. In the present study, we have utilized the AutoDock plugin that can be incorporated in PyMOL, to analyze the binding sites and prepare the input parameters for AutoDock Vina runs. The grid for blind docking runs was generated based on the available data. Earlier reports demonstrated that the Thumb region is important for trimer stability and efflux as demonstrated by P223G mutation^34^. The docking grid generated covers the periplasmic region of *Ec*AcrB except the thumb region and inner membrane region. The receptor structural information required by the program (the pdbqt files) were generated using PyMOL with the AutoDock plugin, and the ligand pdbqt files were generated by utilizing scripts included in the Molecular Graphics Laboratory (MGL) tools.6^35^.

### Molecular Dynamics simulations

Initial binding orientations of bound ligands were obtained from molecular docking studies with AcrB efflux pump. Reduced AcrB model generated based on residue list provided in earlier literature^19^. Force field parameters for ligands were created with the Antechamber program^36^ from the Amber14 package using the General Amber Force Field (GAFF) and AM1-BCC^37,38^, partial charges. All molecular dynamics simulations were performed using the Amber14 molecular dynamics package and the Amber99SB^39^ force field. Energy minimization and MD was carried out with PMEMD. A total of four minimization steps were executed prior to the heating step. For minimization, restraints employed a harmonic force constant of 100.0 kcal/(mol **·** Å^2^). First, each ligand molecule was minimized with restraints applied to all protein heavy atoms. Next, the total system (protein and ligand) was minimized with no restraints. Each minimized system was then inserted in a water box of TIP3P water, which extended at least 10 Å away from any given protein atom, and neutralized by adding counter ions. Solvent molecules were then minimized while restraining all protein and ligand heavy atoms. Finally, all restraints were removed and the total system (protein, ligand, and solvent) was minimized. Each energy minimization procedure used the steepest descent method for the first 3000 steps and then conjugated gradient method for the subsequent 2000 steps. After energy minimization, the system was slowly heated from 0 to 300.0 K over 400 ps in the NPT ensemble under 1 atm pressure to equilibrate the solvent. A harmonic restraint weight of 5.0 kcal/(mol **·** Å2) was applied to all heavy atoms in this first 400 ps. An additional MD equilibration of 200 ps was performed with a decreased restraint weight of 2.0 kcal/(mol **·** Å2), followed by a final equilibration lasting 200 ps with no restraints. Production MD simulations of 25 ns each were carried out without any restraint at a temperature of 300.0 K and a pressure of 1 atm. Unless otherwise noted, all MD simulations used a time step of 2 fs, periodic boundary conditions were employed, and all electrostatic interactions were calculated using the particle mesh Ewald (PME) method^40^. A 10.0 Å cutoff was used to calculate the direct space sum of PME, and bond lengths involving bonds to hydrogen atoms were constrained using the SHAKE algorithm^41^. The coordinates were stored every 10 ps for each production MD run. Ligand RMSD was measured by utilizing the Amber analyses tool PTRAJ. Ligand RMSD values were computed by aligning the protein backbone from MD snapshots and using the “nofit” option for ligand molecule. These amber trajectories were converted to Desmond molecular software readable formats (.cms) for further analyses. Simulation Interactions Diagram method incorporated in Desmond module^42^ of Schrodinger was used to read the converted trajectories and generate ligand interaction diagrams. Residual decomposition analyses by MM/GBSA method was performed using 1500 snapshots (every 10^th^ snapshot from 10000 to 25000 ps).

### *In vivo* pharmacokinetic studies of compound 12 and 15 in BALB/c mice

#### Intravenous Pharmacokinetics

These studies were performed as per CPCSEA, Govt. of India approved protocols (Registration number 1610/RO/c/12/CPCSEA), and also following approval by the Institutional Animals Ethics Committee (IAEC).

The test substances were formulated in the vehicle DMSO: PEG400: Ethanol (absolute): Water at ratio of 10:40:20:30 (v/v). The required quantity of test compound was weighed, transferred into a vial and dissolved using the appropriate volumes of DMSO, PEG400, Ethanol (absolute) and sterile water in a sequential manner until a clear solution was obtained. The test substance was administered intravenously as a bolus via the tail vein at a dose volume of 5 ml/kg.

#### Oral Pharmacokinetics

The test substances were formulated in 10% DMSO in 0.25% carboxy methyl cellulose. 40 mg of the test compounds were weighed and transferred into a mortar followed by the addition of 0.4 ml of DMSO to dissolve the compound. Then 3.6 ml of 0.25% of CMC was added in aliquots to the DMSO solution, with constant grinding using a pestle until a uniform suspension was obtained. The final concentration of the compound was 10 mg/ml. The compounds were administered orally, by gavage, at a dose volume of 10 ml/kg.

#### Sampling

At 5 min, 15 min, 30 min, 1 h, 2 h, 4 h, 8 h, and 24 h time points, and prior to sampling, mice were anaesthetized with isoflurane. From each mouse, two blood samples were drawn; the 1^st^ was non-terminal (0.1 ml) and the 2^nd^ terminal, by retro-orbital plexus puncture using heparanised capillary tubes. Blood was collected in 2 ml Eppendorf tubes containing 0.01 ml of 10% K_2_EDTA as anticoagulant, mixed gently and placed in ice before centrifugation. Blood was centrifuged at 10,000 rpm for 5 min, and plasma was harvested and stored at −80 °C until bioanalysis.

#### Bioanalysis

Ultrafast liquid chromatography mass spectrometric method was used for the estimation of test compounds in pre-clinical study samples. The analysis was performed using an API 4000 LC-MS/MS system. The interface used with the API 4000 LC-MS/MS was a Turbo Ion spray. The positive ions were measured in MRM mode. The analyte was extracted using a solid-phase extraction procedure. The data was acquired by and integrated using the Applied Biosystems “Analyst” version 1.5.2 software. Linear regression, with 1/x^2^ weighting was used for calibration.

#### PK Analysis

Non-compartmental (NCA) PK analysis was performed using MS Excel. The linear trapezoidal rule was used to estimate the AUC. The NCA analysis was performed using the following equations (3) on mean concentration time data:

$${\rm{AUC}}={\rm{\Sigma }}[(\text{Ci1}+\text{Ci2})/2]\ast [{\rm{ti2}}-{\rm{ti1}}]$$

$${\rm{AUMC}}={\rm{\Sigma }}[(({\rm{Ci1}}\ast {\rm{ti1}})+({\rm{Ci2}}\ast {\rm{ti2}}))/2)\ast ({\rm{ti2}}-{\rm{ti1}})]$$

$${{\rm{AUC}}}_{{\rm{t}}-\infty }={{\rm{Cl}}}_{{\rm{ast}}}/{{\rm{K}}}_{{\rm{el}}}$$

$${{\rm{AUC}}}_{0-\infty }={{\rm{AUC}}}_{0-{\rm{t}}}+{{\rm{AUC}}}_{{\rm{t}}-\infty }$$

$${{\rm{A}}{\rm{U}}{\rm{C}}}_{{\rm{e}}{\rm{x}}{\rm{t}}{\rm{r}}{\rm{a}}{\rm{p}}{\rm{o}}{\rm{l}}{\rm{a}}{\rm{t}}{\rm{e}}{\rm{d}}}({\rm{ \% }})=[{{\rm{A}}{\rm{U}}{\rm{C}}}_{{\rm{t}}-{\rm{\infty }}}/{{\rm{A}}{\rm{U}}{\rm{C}}}_{0-{\rm{\infty }}}]\ast 100$$

where, Ci1 and Ci2 are the concentrations at times ti1 and ti2, respectively.

$${{\rm{K}}}_{{\rm{el}}}=\,\mathrm{ln}(\text{Cx}/\text{Cy})/({\rm{ty}}-{\rm{tx}})$$

where, K_el_ is the first order elimination rate constant, ln is the natural logarithm, Cx and Cy are the first and last concentrations, respectively on the linear portion of the terminal phase of the concentration-time profile, and ty and tx are the corresponding time points.

$${{\rm{T}}}_{1/2}=0.693/{{\rm{K}}}_{{\rm{el}}}$$

$${\rm{Cl}}={\rm{Doseiv}}/{{\rm{AUC}}}_{0-\infty }$$

$${{\rm{V}}}_{{\rm{ss}}}=({{\rm{AUMC}}}_{0-\infty }/{{\rm{AUC}}}_{0-\infty })\ast ({\rm{Doseiv}}/{{\rm{AUC}}}_{0-\infty })$$

C_max_ and t_max_ were visually estimated from the mean oral conc-time profile

$${\rm{F}}( \% )=(\text{AUCpo}/\text{AUCiv})\ast (\text{Doseiv}/\text{Dosepo})\ast 100$$

### Neutropenic Thigh Infection Efficacy Studies

In the first study (Study No: TI-2014-007), neutropenic mice were infected with 10^7^ CFU/ml of *E. coli* ∆*acrB* JW0451-2 in the thigh of BALB/c mice. Two hours post infection mice were treated orally with single doses of vehicle, ciprofloxacin (100 mg/kg), compound 15 (10, 30 and 100 mg/kg). Efficacy was assessed at 8 hours post infection (6 hours post treatment) by enumerating bacteria from the thigh.

In the second experiment (Study No: TI-2014-008) neutropenic mice were infected with 10^7^ CFU/ml of *E. coli* strains BW25113 and *E. coli* ∆*acrB* JW0451-2. Two hour post infection, mice were treated orally with single doses of vehicle, ciprofloxacin (100 mg/kg) and compound 15 (30 and 100 mg/kg). Efficacy was assessed at 8 hours post infection (6 hours post treatment) by estimating the bacterial counts in thighs.

In the third experiment (Study No: TI-2014-023) neutropenic mice were infected with 10^7^ CFU/ml of a clinical isolate of *E. coli* (Strain No. 371, sensitive to ciprofloxacin; MIC 0.02 μg/ml), obtained from St. John’s Medical Hospital, Bengaluru. Two hours post infection, mice were treated orally with single doses of vehicle, ciprofloxacin (100 mg/kg), compound 15 (100 mg/kg), and compound 12 (100 mg/kg). Efficacy was assessed at 8 hours post infection (6 hours post treatment) by estimating the bacterial counts in thighs.

### General Chemical methods and synthesis

All commercial reagents and solvents were used without further purification. Analytical thin-layer chromatography (TLC) was performed on SiO_2_ plates on alumina. Visualization was accomplished by UV irradiation at 254 and 220 nm. Purity of all final derivatives for biological testing was confirmed to be >95% as determined using the following conditions. Evaporations were carried out by rotary evaporation in vacuo and work up procedures were carried out after removal of residual solids by filtration; temperatures are quoted as °C; operations were carried out at room temperature, that is typically in the range 18 to 26 °C and without the exclusion of air unless otherwise stated, or unless the skilled person would otherwise work under an inert atmosphere; column chromatography (by the flash procedure) was used to purify compounds and was performed on Merck Kiesel gel silica (Art. 9385) unless otherwise stated; in general, the course of reactions were followed by TLC, HPLC, or LC/MS and reaction times are given for illustration only; yields are given for illustration only and are not necessarily the maximum attainable; the structure of the end products of the invention was generally confirmed by NMR and mass spectral techniques. Proton magnetic resonance spectra were generally determined in DMSO d6 unless otherwise stated, using a Bruker DRX 300 spectrometer or a Bruker DRX-400 spectrometer, operating at a field strength of 300 MHz, or 400 MHz, respectively. In cases where the NMR spectrum is complex, only diagnostic signals are reported. Chemical shifts are reported in parts per million downfield from tetramethylsilane as an external standard (* scale) and peak multiplicities are shown thus: s, singlet; d, doublet; dd, doublet of doublets; dt, doublet of triplets; dm, doublet of multiplets; t, triplet, m, multiplet; br, broad. Fast atom bombardment (FAB) mass spectral data were generally obtained using a Platform spectrometer (supplied by Micromass) run in electrospray and, where appropriate, either positive ion data or negative ion data were collected or using Agilent 1100 series LC/MS equipped with Sedex 75ELSD, and where appropriate, either positive ion data or negative ion data were collected. The lowest mass major ion is reported for molecules where isotope splitting results in multiple mass spectral peaks (for example when chlorine is present). Reverse Phase HPLC was carried out using YMC Pack ODS AQ (100 × 20 mmID, S 5 Å particle size, 12 nm pore size) on Agilent instruments; each intermediate was purified to the standard required for the subsequent stage and was characterized in sufficient detail to confirm that the assigned structure was correct; purity was assessed by HPLC, TLC, or NMR and identity was determined by infrared spectroscopy (IR), mass spectroscopy or NMR spectroscopy as appropriate.

### Compound 7: N-(5-Methyl-4-phenylthiazol-2-yl)-5-nitrothiophene-2-carboxamide (CAS: 324759-10-6,)

#### Step-1: Synthesis of 5-methyl-4-phenylthiazol-2-amine (7a)

To a stirred solution of 2-bromopropiophenone (10 g, 46.93 mM, 1 eq) in ethanol (200 mL), Thiourea (3.75 g, 49.27 mM, 1.05 equiv) was added at 25 °C. The reaction mixture was heated at 85 °C for 3 h. After completion of the reaction, the reaction mixture was cooled to 25 °C and concentrated in vacuo to obtain crude product. The crude product was diluted with water (330 mL) and saturated Na_2_CO_3_ (16 mL) and stirred for 10 min to obtained solid which was filtered and washed with water and dried to give 8.6 g (96.52%) of 5-methyl-4-phenylthiazol-2-amine (Figure S10) as pale yellow solid. ^1^H NMR (300 MHz, DMSO-d_6_) δ = 7.56–7.53 (m, 2H), 7.40–7.36 (m, 2H), 7.29 = 7.26 (m, 1H), 6.77 (s, 2H), 2.31 (s, 3H). LCMS: Calculated for C_10_H_10_N_2_S = 190.26, Observed = 191.0 (M + H).

#### Step-2: Synthesis of N-(5-methyl-4-phenylthiazol-2-yl)-5-nitrothiophene-2-carboxamide

To a stirred solution of 5-Nitro-2-thiophencarboxylic acid (0.91 g, 5.25 mM, 1 eq) in DMF (10 mL), Diisopropylethylamine (1.7 g, 13.12 mmol, and 2.5eq) was added, followed by HATU (1.99 g, 5.25 mmol, 1equiv) at 0 °C and stirred for 30 min. After 30 min 5-methyl-4-phenylthiazol-2-amine (1 g, 5.25 mmol, 1 eq) was added and the reaction mixture was stirred at 25 °C for 12 h. The reaction mixture was poured in to ice water and extracted with ethyl acetate and the combined organic layer was dried over a Na_2_SO_4_, filtered and concentrated to obtained crude product which was purified by column chromatography using neutral alumina, eluent 5-10% ethyl acetate in pet.ether to give N-(5-methyl-4-phenylthiazol-2-yl)-5-nitrothiophene-2-carboxamide (**compound 7**) as amorphous red powder (Figure S11).

^1^H NMR (400 MHz, DMSO-d_6_) δ = 11.99 (bs, 1H), 7.58 (d, 1H, J = 4.4 Hz), 7.43-7.41 (m, 2H), 7.34-7.30 (m, 2H), 7.26-7.23 (m, 2H) 2.54 (s, 3H). LCMS: Calculated for C_15_H_11_N_3_O_3_S_2_ = 345.39, Observed = 346.2 (M + H). HPLC = 95.79% (HPLC Column: Phenomenex Gemini-NX C18 (150 × 4.6 mm) 5 micron, Mobile Phase A: 10 mM Ammonium acetate in water, Mobile Phase B: Acetonitrile.

### Compound 8: N-(5-Methyl-4-phenylthiazol-2-yl)-5-(trifluoromethyl) thiophene-2-carboxamide (CAS: 2147485-13-8)

To a stirred solution of 5-(trifluoromethyl)thiophene-2-carboxylic acid (0.93 g, 4.73 mM, 1 equiv) in DMF (9 mL), Diisopropylethylamine (1.52 g, 11.82 mmol, 2.5 eq) was added, followed by HATU (1.8 g, 4.73 mmol, 1 equiv) at 0 °C and stirred for 30 min. After 30 min, 5-methyl-4-phenylthiazol-2-amine (0.9 g, 4.73 mmol, 1 equiv) was added and the reaction mixture was stirred at 60 °C for 12 h. The reaction mixture was poured in to ice water when a solid precipitated out. The solid was filtered and dried. The crude product was purified by column chromatography using neutral alumina, eluent 5-10% ethyl acetate in pet ether to give 0.18 g of N-(5-methyl-4-phenylthiazol-2-yl)-5-(trifluoromethyl) thiophene-2-carboxamide (Figure S12) as pale yellow amorphous powder.

^1^H NMR (400 MHz, DMSO-d_6_) δ = 11.56 (bs, 1H), 7.46-7.44 (m, 2H,), 7.33-7.27 (m, 3H), 7.25-7.23 (m, 1H), 7.21-7.17 (m, 1H) 2.54 (s, 3H). LCMS: Calculated for C_16_H_11_F_3_N_2_OS_2_ = 368.39, Observed = 369.0. HPLC = 97.71% (HPLC Column: Phenomenex Gemini-NX C18 (150 × 4.6 mm) 5micron, Mobile Phase A: 10 mM Ammonium acetate in water, Mobile Phase B: Acetonitrile.

### Compound 9: N-(4-(4-Fluorophenyl)-5-methylthiazol-2-yl)-5-nitrothiophene-2-carboxamide (CAS: 796081-45-3)

**Compound 9** was procured from the commercial source (Enamine advanced HTS collection) and used for the screening purpose.

### Compound 10: N-(5-((dimethylamino)methyl)-4-(4-fluorophenyl)thiazol-2-yl)-5-nitro thiophene-2-carboxamide

**Compound 10** was synthesized using scheme and procedure analogous to **compound 7** from commercially available 5-[(dimethylamino)methyl]-4-(4-fluorophenyl)-1,3-thiazol-2-amine (**CAS: 955327-92-1**) and 5-Nitro-2-thiophencarboxylic acid.

^1^H NMR (400 MHz, DMSO-d_6_) δ = 8.15-8.20 (m, 2H), 7.71 (bs, 2H), 7.32-7.34 (m, 2H), 3.68 (bs, 2H), 2.25 (s, 6H). LCMS: Calculated for C_17_H_15_FN_4_O_3_S_2_ = 406.45 Observed = 406.8. HPLC = 93.49% (HPLC Column: Phenomenex Gemini-NX C18 (150 × 4.6 mm) 5micron, Mobile Phase A: 10 mM Ammonium acetate in water, Mobile Phase B: Acetonitrile.

### Compound 11: N-(4-(3,4-Difluorophenyl) thiazol-2-yl)-5-nitrothiophene-2-carboxamide (CAS: 954010-93-6)

**Compound 11** was synthesized using scheme and procedure analogous to **compound 7** from commercially available 2-amino-4-(3,4-difluorophenyl) thiazole (**CAS: 175135-32-7**, Alfa Aesar) and 5-Nitro-2-thiophencarboxylic acid.

^1^H NMR (400 MHz, DMSO-d_6_) δ = 13.38 (bs, 1H), 8.26-8.22 (m, 2H,), 7.99-7.94 (m, 1H), 7.87 (s, 1H), 7.80 (s, 1H) 7.56-7.49 (s, 1H). LCMS: Calculated for C_14_H_7_F_2_N_3_O_3_S_2_ = 367.34, Observed = 368.0. HPLC = 96.11% (HPLC Column: ATLANTIS d C 18 (250 × 4.6) mm, 5% m Mobile phase:A:0.1% TFA in water. Mobile phase: B: Acetonitrile.

### Compound 12: N-(4-(2,4-difluorophenyl)thiazol-2-yl)-5-nitrothiophene-2-carboxamide (CAS: 953943-03-8)

**Compound 12** was synthesized using scheme and procedure analogous to **compound 7** from commercially available 2-amino-4-(2,4-difluorophenyl) thiazole (**CAS: 105512-80-9**, Alfa Aesar) and 5-Nitro-2-thiophencarboxylic acid.

^1^H NMR (400 MHz, DMSO-d_6_) δ = 8.27-8.22 (m, 2H,), 8.11-8.09 (m, 1H), 7.64 (s, 1H), 7.43-7.38 (m, 1H) 7.25-7.21 (m, 1H). LCMS: Calculated for C_14_H_7_F_2_N_3_O_3_S_2_ = 367.34, Observed = 368.0. HPLC = 96.94% (HPLC Column: ATLANTIS d C18 (250 × 4.6) mm, 5% m Mobile phase:A:0.1% TFA in water. Mobile phase: B: Acetonitrile.

### Compound 13: N-(4-(3-Fluoro-4-methylphenyl)thiazol-2-yl)-5-nitrothiophene-2-carboxamide

**Compound 13** was synthesized using scheme and procedure analogous to **compound 7** from commercially available 2-Amino-4-(4′-fluoro-3′-methyl) phenylthiazole (**CAS: 3830-48-6**, Combi- Block) and 5-Nitro-2-thiophencarboxylic acid.

^1^H NMR (400 MHz, DMSO-d_6_) δ = 13.35 (bs, 1H), 8.23-8.28 (m, 2H), 7.83 (s, 1H), 7.68-7.71 (m, 2H), 7.35-7.39 (m, 1H), 2.27 (s, 3H). LCMS: Calculated for C_15_H_10_FN_3_O_3_S_2_ = 363.38, Observed = 364.0. HPLC = 94.24% (HPLC Column: Phenomenex Gemini-NX C18 (150 × 4.6 mm) 5 micron, Mobile Phase A: 10 mM Ammonium acetate in water, Mobile Phase B:

### Compound 14: N-(4-(4-cyanophenyl) thiazol-2-yl)-5-nitrothiophene-2-carboxamide (CAS: 2147485-11-6)

**Compound 14** was synthesized using scheme and procedure analogous to **compound 7** from commercially available 4-(2-Amino-1,3-thiazol-4-yl) benzonitrile (**CAS: 436151-85-8**, Combi- Block) and 5-Nitro-2-thiophencarboxylic acid.

^1^H NMR (400 MHz, DMSO-d6) δ = 13.42 (bs, 1H), 8.25-8.21 (m, 2H, J = 8 Hz), 8.12 (d, 2H, J = 8 Hz), 8.06 (s, 1H), 7.92 (d, 1H, J = 8.4 Hz). LCMS: Calculated for C_15_H_8_N_4_O_3_S_2_ = 356.37, Observed = 357.0. HPLC = 99.21% (HPLC Column: Welchrom C18 (250 * 4.6) mm 5 micron Mobile phase A: 0.1%TFA in Water, Mobile phase B: CAN.

### Compound 15: N-(4-(4-Fluorophenyl)thiazol-2-yl)-5-nitrothiophene-2-carboxamide (CAS: 308292-78-6)

**Compound 15** (**CAS: 308292-78-6**) was procured from the commercial source (Life chemicals HTS compounds) and used for the screening purpose.

### Compound 16: 5-Nitro-N-(4-(4-(trifluoromethoxy)phenyl)thiazol-2-yl)thiophene-2-carboxamide (CAS: 2147485-27-4)

**Compound 16** was synthesized using scheme and procedure analogous to **compound 7** from commercially available 2-Amino-4-[4-(trifluoromethoxy) phenyl]-1,3-thiazole (**CAS: 436151-95-0**, Apollo Scientific Fluorine Chemicals) and 5-Nitro-2-thiophencarboxylic acid.

^1^H NMR (400 MHz, DMSO-d_6_) δ = 13.40 (bs, 1H), 8.25-8.22 (m, 2H,), 8.07-8.04 (m, 2H), 7.85 (s, 1H), 7.46-7.44 (m, 2H). LCMS: Calculated for C_15_H_8_F_3_N_3_O_4_S_2_ = 415.36, Observed = 415.8, HPLC = 98.39% (HPLC Column: ATLANTIS dC18 (250 × 4.6 mm) 5 micron, Mobile Phase A: 0.1% TFA in water, Mobile Phase B: Acetonitrile.

### Compound 17: 5-Nitro-N-(4-(6-(trifluoromethyl)pyridin-3-yl)thiazol-2-yl)thiophene-2-carboxamide

**Compound 17** was synthesized using scheme and procedure analogous to **compound 7** from commercially available 4-[5-(trifluoromethyl) pyridin-2-yl]-1,3-thiazol-2-amine (**CAS**: **1595798-43-8**, UORSY Building Blocks Library) and 5-Nitro-2-thiophencarboxylic acid.

^1^H NMR (400 MHz, DMSO-d_6_) δ = δ 13.53 (bs, 1H), 9.35 (s, 1H), 8.55-8.58 (m, 1H), 8.20-8.29 (m, 3H), 8.02 (d, J = 8.00 Hz, 1H). LCMS: Calculated for C_14_H_7_F_3_N_4_O_3_S_2_ = 400.35, Observed = 401. HPLC = 98.954% (HPLC Column: Phenomenex Gemini-NX C18 (150 × 4.6 mm) 5 micron, Mobile Phase A: 10 mM Ammonium acetate in water, Mobile Phase B: Acetonitrile.

### Compound 18: N-(4-(4-fluoro-2-methylphenyl)thiazol-2-yl)-5-nitrothiophene-2-carboxamide (CAS: 2147485-32-1)

**Compound 18** was synthesized using scheme and procedure analogous to **compound 7** from commercially available 4-(4-fluoro-2-methylphenyl)-1,3-thiazol-2-amine (**CAS**: **23178-08-7**, UORSY Building Blocks Library) and 5-Nitro-2-thiophencarboxylic acid

^1^H NMR (400 MHz, DMSO-d_6_) δ = 13.33 (bs, 1H), 8.22-8.25 (m, 2H), 7.64 (s, 1H), 7.41 (s, 1H), 7.09-7.19 (m, 2H), 2.45 (s, 3H). LCMS: Calculated for C_15_H_10_FN_3_O_3_S_2_ = 363.38, Observed = 363.8. HPLC = 98.81% (HPLC Column: Phenomenex Gemini-NX C18 (150 × 4.6 mm) 5 micron, Mobile Phase A: 10 mM Ammonium acetate in water, Mobile Phase B: Acetonitrile.

### Compound 19: N-(4-(4-fluoro-3-methoxyphenyl)thiazol-2-yl)-5-nitrothiophene-2-carboxamide (CAS: 2147485-25-2)

**Compound 19** was synthesized using scheme and procedure analogous to **compound 7** from commercially available 4-(4-fluoro-3-methoxyphenyl)-1,3-thiazol-2-amine (**CAS: 187847-98-9**, UORSY Building Blocks Library) and 5-Nitro-2-thiophencarboxylic acid.

^1^H NMR (400 MHz, DMSO-d_6_) δ = 13.37 (bs, 1H), 8.23-8.27 (m, 2H), 7.81 (s, 1H), 7.70 (d, J = 8.60 Hz, 1H), 7.52 (s, 1H), 7.27-7.32 (m, 1H), 3.92 (s, 3H). LCMS: Calculated for C_15_H_10_FN_3_O_4_S_2 = _379.38, Observed = 380.0. HPLC = 98.339% (HPLC Column: Phenomenex Gemini-NX C18 (150 × 4.6 mm) 3 micron, Mobile Phase A: 10 mM Ammonium acetate in water, Mobile Phase B: Acetonitrile.

### Compound 20: N-(4-(4-chloro-3-methoxyphenyl)thiazol-2-yl)-5-nitrothiophene-2-carboxamide (CAS: 2147485-35-4)

#### Step 1: Synthesis of 1-chloro-4-(1-ethoxyvinyl)-2-methoxybenzene (20a)

A mixture of 4-bromo-1-chloro-2-methoxybenzene (10 g, 45.14 mM, 1.0 equiv), tributyl(1-ethoxyvinyl)stannane (17.93 g, 49.66 mM, 1.1 equiv) was taken in dry DMF at 20 °C, tetrakis(triphenylphosphine)palladium (0) (2.6 g, 2.25 mM, 0.05 equiv) was added under N2 atmosphere and heated to 80 °C for 1 h. After completion of the reaction (monitored by TLC), the reaction mixture was diluted with EtOAc (100 mL) and filtered through celite, washed with EtOAc. The combined filtrate was washed with water, brine and dried over anhy.Na_2_SO_4_. Filtered and the solvent was evaporated under reduced pressure to yield the crude product. It was purified by silica gel (60–120 mesh) column chromatography using petroleum ether: ethyl acetate (90:10) to afford 8.5 g (89%) of 1-chloro-4-(1-ethoxyvinyl)-2-methoxybenzene, **20a** (Figure S13) as pale brown liquid which was used for the next step.

#### Step 2: Synthesis of 2-bromo-1-(4-chloro-3-methoxyphenyl)ethan-1-one (20b)

To a stirred solution of 1-chloro-4-(1-ethoxyvinyl)-2-methoxybenzene, **20a** (8.5 g, 39.96 mM, 1.0 equiv) in THF: water (110 mL: 4 mL), N-bromosuccinimide (12.8 g, 71.94 mM, 1.8 equiv) was added at 25 °C and stirred for 24 h. After completion of the reaction (monitored by TLC), the reaction mixture was filtered through celite, washed with EtOAc. The combined filtrated was concentrated under reduced pressure and the residue was taken in MTBE, washed with water, brine and dried over anhy.Na_2_SO_4_. Filtered and the solvent was evaporated under reduced pressure to yield 10 g (**crude**) of 2-bromo-1-(4-chloro-3-methoxyphenyl)ethan-1-one, **20b** as yellow liquid. The crude product was taken as such to the next step without purification. GCMS: Calculated for C_9_H_8_BrClO_2_ = 263.52, Observed = 263.9.

#### Step 3: Synthesis of 4-(4-chloro-3-methoxyphenyl)thiazol-2-amine (20c)

To a stirred solution of 2-bromo-1-(4-chloro-3-methoxyphenyl)ethan-1-one, **20b** (crude, 10 g) in EtOH (10 mL) at 25 °C, thiourea (2.9 g) was added and stirred for 30 min. After completion of the reaction (monitored by TLC), the reaction mixture was concentrated under reduced pressure to yield the crude product. It was stirred with aq. Ammonia (25wt % in water) at 25 °C for 30 min. The solid thus obtained was filtered, washed with water and dried to afford 6.38 g (crude) of 4-(4-chloro-3-methoxyphenyl)thiazol-2-amine, **20c** as pale yellow solid. It was taken as such to the next step without purification. LCMS: Calculated for C_10_H_9_ClN_2_OS = 240.71, Observed = 241.0

#### Step 4: Synthesis of N-(4-(4-chloro-3-methoxyphenyl)thiazol-2-yl)-5-nitrothiophene-2-carboxamide (compound 20)

To a stirred solution of 5-Nitro-2-thiophencarboxylic acid (0.45 g, 2.59 mM, 1.0 equiv) in dry dichloromethane (15 mL), triethylamine (0.7 mL, 4.48 mM, and 4.0 equiv) was added, followed by HATU (0.94 g, 2.48 mM, 1.2 equiv) at 0 °C and stirred for 30 min. Then 4-(4-chloro-3-methoxyphenyl)thiazol-2-amine, **20c** (0.5 g, 2.07 mmol, 1.0 equiv) was added and the reaction mixture was stirred at 25 °C for 3 h. After completion of the reaction (monitored by TLC & LCMS), the reaction mixture was diluted with dichloromethane (10 mL), washed with aqueous 10% NaHCO_3_ solution, brine and dried over anhy.Na_2_SO_4_. Filtered and the solvent was evaporated under reduced pressure to yield the crude product. It was stirred with methanol at 25 °C for 30 min, the solid thus obtained was filtered, washed with methanol and dried to yield 0.676 g (82%) of N-(4-(4-chloro-3-methoxyphenyl)thiazol-2-yl)-5-nitrothiophene-2-carboxamide as yellow amorphous powder. ^1^H NMR (400 MHz, DMSO-d_6_) δ = 13.39 (bs, 1H), 8.19-8.22 (m, 2H), 7.88 (s, 1H), 7.67 (s, 1H), 7.47-7.55 (m, 2H), 3.93 (s, 3H). LCMS: Calculated for C_15_H_10_ClN_3_O_4_S_2_ = 395.83, Observed = 396.0. HPLC = 97.48% (HPLC Column: Phenomenex Gemini-NX C18 (150 × 4.6 mm) 5 micron, Mobile Phase A: 10 mM Ammonium acetate in water, Mobile Phase B: Acetonitrile).

### Compound 21: N-(4-(4-Chloro-3-fluorophenyl)thiazol-2-yl)-5-nitrothiophene-2-carboxamide (CAS: 2147485-26-3)

**Compound 21** was synthesized using scheme and procedure analogous to **compound 7** from commercially available 4-(4-chloro-3-fluorophenyl)-1,3-thiazol-2-amine (**CAS: 1702084-35-2**, UORSY Building Blocks Library) and 5-Nitro-2-thiophencarboxylic acid.

^1^H NMR (400 MHz, DMSO-d_6_) δ = 13.42 (bs, 1H), 8.23-8.26 (m, 2H), 7.94-7.97 (m, 2H), 7.82-7.85 (m, 1H), 7.67-7.71 (m, 1H). LCMS: Calculated for C_14_H_7_ClFN_3_O_3_S_2_ = 383.8, Observed = 384.0. HPLC = 96.93% (HPLC Column: ATLANTIS dC18 (250 × 4.6), 5micron, Mobile Phase A: 0.1% TFA in water, Mobile Phase B: Acetonitrile.

### Compound 22: N-(4-(4-chloro-3-ethoxyphenyl)thiazol-2-yl)-5-nitrothiophene-2-carboxamide (CAS: 2147485-36-5)

#### Step 1: Synthesis of 4-bromo-1-chloro-2-ethoxybenzene (22a)

A mixture of 5-bromo-2-chlorophenol (2 g, 9.64 mM, 1.0 equiv), K_2_CO_3_ (2.66 g, 19.28 mM, 2.0 equiv), iodoethane (1.95 g, 12.493 mM, 1.3 equiv) and DMF (10 mL) was stirred at 55 °C for 3 h. After completion of the reaction (monitored by TLC), the reaction mixture was poured into crushed ice and extracted with EtOAc. The combined organic extracts were washed with water, brine and dried over Na_2_SO_4_. Filtered and evaporated under reduced pressure to yield the crude product. It was purified by silica gel (60–120 mesh) column chromatography using petroleum ether: ethyl acetate (90:10) to afford 2 g (88%) of 4-bromo-1-chloro-2-ethoxybenzene, **22a** (Figure S14) as colourless liquid.

#### Step 2: Synthesis of 1-(4-chloro-3-ethoxyphenyl)ethan-1-one (22b)

To stirred solution of toluene (5 mL) at −78 °C, n-Butyl lithium (2.5 M in hexane, 1.2 mL, 2.97 mMl, 1.4 equiv) was added, followed by the addition of 4-bromo-1-chloro-2-ethoxybenzene, **22a** (0.5 g, 2.12 mM, 1.0 equiv) in toluene (5 mL) and stirred for 1 h. Then N,N-Dimethylacetamide (0.22 mL, 2.33 mM, 1.1 equiv) was added dropwise at −78 °C and stirred for 30 min. After completion of the reaction (monitored by TLC), the reaction mixture was quenched with saturated NH_4_Cl and extracted with MTBE. The combined organic extracts were washed with water, brine and dried over a Na_2_SO_4_. Filtered and the solvent was evaporated under reduced pressure to afford 0.42 g (**crude**) of 1-(4-chloro-3-ethoxyphenyl)ethan-1-one, **22b** as colorless liquid. It was taken as such to the next step without purification.

#### Step 3: Synthesis of 2-bromo-1-(4-chloro-3-ethoxyphenyl)ethan-1-one (22c)

To a stirred solution of 1-(4-chloro-3-ethoxyphenyl)ethan-1-one, **22b** (crude, 0.4 g, 2.01 mM, 1.0 equiv) in HBr in AcOH (30 wt% in AcOH, 4 mL) at 0 °C, Bromine (0.08 mL, 1.61 mM, 0.8 equiv) was added dropwise and stirred for 1 h. After completion of the reaction (monitored by TLC), the reaction mixture was poured into crushed ice and extracted with MTBE. The combined organic extracts were washed with aqueous 10% NaHCO_3_ solution, brine and dried over anhy.Na_2_SO_4_. Filtered and the solvent was evaporated under reduced pressure to afford 0.5 g (crude) of 2-bromo-1-(4-chloro-3-ethoxyphenyl)ethan-1-one, **22c** as gummy solid. It was taken as such to the next step without purification.

#### Step 4: Synthesis of 4-(4-chloro-3-ethoxyphenyl)thiazol-2-amine (22d)

To a stirred solution of 2-bromo-1-(4-chloro-3-ethoxyphenyl)ethan-1-one, **22c** (**crude**, 0.5 g) in EtOH (80 mL) at 25 °C, thiourea (0.13 g) was added and stirred for 1 h. After completion of the reaction (monitored by TLC), the reaction mixture was concentrated under reduced pressure to yield the crude product. It was stirred with aq. Ammonia (25 wt% in water) at 25 °C for 30 min. The solid thus obtained was filtered, washed with water and dried to afford 0.11 g (crude) of 4-(4-chloro-3-ethoxyphenyl)thiazol-2-amine, **22d** as yellow amorphous powder. It was taken as such to the next step without purification.

#### Step 5: Synthesis of N-(4-(4-chloro-3-ethoxyphenyl)thiazol-2-yl)-5-nitrothiophene-2-carboxamide (compound 22)

To a stirred solution of 5-Nitro-2-thiophencarboxylic acid (0.1 g) in dry dichloromethane (10 mL), was added triethylamine (0.7 mL) followed by HATU (0.3 g) at 0 °C and stirred for 30 min. Then 4-(4-chloro-3-ethoxyphenyl)thiazol-2-amine, **22d** (0.11 g) was added and the reaction mixture was stirred at 25 °C for 3 h. After completion of the reaction (monitored by TLC & LCMS), the reaction mixture was diluted with dichloromethane (10 mL), washed with aqueous 10% NaHCO_3_ solution, brine and dried over anhy.Na_2_SO_4_. Filtered and the solvent was evaporated under reduced pressure to yield the crude product. It was stirred with methanol at 25 °C for 30 min, the solid thus obtained was filtered, washed with methanol and dried to yield 0.015 g of N-(4-(4-chloro-3-ethoxyphenyl)thiazol-2-yl)-5-nitrothiophene-2-carboxamide, as yellow amorphous powder. ^1^H NMR (400 MHz, DMSO-d_6_) δ = 13.37 (bs, 1H), 8.23-8.27 (m, 2H), 7.90 (s, 1H), 7.67-7.67 (m, 1H), 7.49-7.55 (m, 2H), 4.20 (q, J = 6.96 Hz, 2H), 1.41 (t, J = 6.96 Hz, 3H). LCMS: Calculated for C_16_H_12_ClN_3_O_4_S_2_ = 409.86, Observed = 409.8. HPLC = 96.797% (HPLC Column: Phenomenex Gemini-NX C18 (150 × 4.6 mm) 5 micron, Mobile Phase A: 10 mM Ammonium acetate in water, Mobile Phase B: Acetonitrile).

### Compound 23: N-(4-(3-methoxy-4-(trifluoromethyl)phenyl)thiazol-2-yl)-5-nitrothiophene-2-carboxamide (CAS: 2147485-37-6)

#### Step 1: Synthesis of N,3-dimethoxy-N-methyl-4-(trifluoromethyl)benzamide (23a)

To a stirred solution of 3-methoxy-4-(trifluoromethyl)benzoic acid (0.2 g, 0.91 mM, 1.0 equiv) in dichloromethane (6 mL) at 25 °C, N,O-Dimethylhydroxylamine Hydrochloride (0.13 g, 1.36 mM, 1.5 equiv), 1-Hydroxybenzotriazole hydrate (0.15 g, 1.08 mM, 1.2 equiv) and triethylamine (0.5 mL, 3.63 mM, 4.0 equiv) were added under N_2_ atmosphere. Then *N*-(3-Dimethylaminopropyl)-*N*′-ethylcarbodiimide hydrochloride (EDCI.HCl) (0.21 g, 1.08 mM, 1.2 equiv) was added at 0^ο^C and stirred at 25 °C for 16 h. After completion of the reaction (monitored by TLC), the reaction mixture was diluted with dichloromethane (100 mL), washed with aqueous 10% NaHCO_3_ solution, brine and dried over anhy.Na_2_SO_4_. Filtered and the solvent was evaporated under reduced pressure to yield the crude product. It was purified by silica gel (60–120 mesh) column chromatography using petroleum ether: ethyl acetate (80:20) to afford 0.23 g (96%) of N,3-dimethoxy-N-methyl-4-(trifluoromethyl)benzamide, **23a** (Figure S15) as colorless liquid. The crude product was used as such for the next step without purification. GCMS: Calculated for C_11_H_12_F_3_NO_3_ = 263.22, Observed = 263.1.

#### Step 2: Synthesis of 1-(3-methoxy-4-(trifluoromethyl)phenyl)ethan-1-one (23b)

To a stirred solution of N,3-dimethoxy-N-methyl-4-(trifluoromethyl)benzamide, **23a** (0.87 g, 3.3 mM, 1.0 equiv) in dry tetrahydrofuran (10 mL), methylmagnesium chloride solution (3 M in THF, 1.5 mL, 4.62 mmol, 1.4 equiv) was added dropwise at 0 °C and stirred for 1 h. After completion of the reaction (monitored by TLC), the reaction mixture was quenched by the slow addition of 1.5 N HCl (10 mL) and extracted with MTBE. The combined organic layers were washed with water, brine and dried over anhy.Na_2_SO_4_. Filtered and the solvent was evaporated under reduced pressure to yield 0.71 g (crude) of 1-(3-methoxy-4-(trifluoromethyl)phenyl)ethan-1-one, **25b** as pale yellow liquid. The crude product was taken as such to the next step without further purification. GCMS: Calculated for C_10_H_9_F_3_O_2_ = 218.18, Observed = 218.0.

#### Step 3: Synthesis of 2-bromo-1-(3-methoxy-4-(trifluoromethyl)phenyl)ethan-1-one (23c)

To a stirred solution of 1-(3-methoxy-4-(trifluoromethyl)phenyl)ethan-1-one, **23b** (0.7 g, 3.2 mM, 1.0 equiv) in HBr in AcOH (30 wt% in AcOH, 7 mL) at 0 °C, Bromine (0.41 mL, 2.56 mM, 0.8 equiv) was added dropwise and stirred for 1 h. After completion of the reaction (monitored by TLC), the reaction mixture was poured into crushed ice and extracted with MTBE. The combined organic extracts were washed with aqueous 10% NaHCO_3_ solution, brine and dried over anhy.Na_2_SO_4_. Filtered and the solvent was evaporated under reduced pressure to afford 0.77 g (**crude**) of 2 2-bromo-1-(3-methoxy-4-(trifluoromethyl)phenyl)ethan-1-one, **25c** as an off-white powder. The crude product was taken as such to the next step without purification. GCMS: Calculated for C_10_H_8_BrF_3_O_2_ = 297.07, Observed = 298.0.

#### Step 4: Synthesis of 4-(3-methoxy-4-(trifluoromethyl)phenyl)thiazol-2-amine (23d)

To a stirred solution of 2-bromo-1-(3-methoxy-4-(trifluoromethyl)phenyl)ethan-1-one, **23c** (0.76 g, 2.55 mM, 1.0 equiv) in EtOH (15 mL) at 25 °C, thiourea (0.195 g, 2.55 mM, 1.0 equiv) was added and stirred for 1 h. After completion of the reaction (monitored by TLC), the reaction mixture was concentrated under reduced pressure to yield the crude product. It was stirred with aq. Ammonia (25 wt% in water) at 25 °C for 30 min. The solid thus obtained was filtered, washed with water and dried to yield 0.5 g (crude) of 4-(3-methoxy-4-(trifluoromethyl)phenyl)thiazol-2-amine, **23d** as yellowish amorphous powder. The product was taken as such to the next step without purification. LCMS: Calculated for C_11_H_9_F_3_N_2_OS = 274.26, Observed = 275.0.

#### Step 5: Synthesis of N-(4-(3-methoxy-4-(trifluoromethyl)phenyl)thiazol-2-yl)-5-nitrothiophene-2-carboxamide

To a stirred solution of 5-Nitro-2-thiophencarboxylic acid (0.126 g, 0.727 mM, 1.0 equiv) in dry dichloromethane (6 mL), triethylamine (0.4 mL, 2.9 mM, and 4.0 equiv) was added, followed by HATU (0.33 g, 0.872 mM, 1.2 equiv) at 0 °C and stirred for 30 min. Then 4-(3-methoxy-4-(trifluoromethyl)phenyl)thiazol-2-amine, **23d** (0.2 g, 0.727 mmol, 1.0 equiv) was added and the reaction mixture was stirred at 25 °C for 16 h. After completion of the reaction (monitored by TLC & LCMS), diluted with dichloromethane (100 mL), washed with aqueous 10% NaHCO_3_ solution, brine and dried over anhy.Na_2_SO_4_. Filtered and the solvent was evaporated under reduced pressure to yield the crude product. It was purified by silica gel (60–120 mesh) column chromatography using petroleum ether: ethyl acetate (60:40) to afford 0.13 g (42%) of N-(4-(3-methoxy-4-(trifluoromethyl)phenyl)thiazol-2-yl)-5-nitrothiophene-2-carboxamide, as an orange amorphous powder. ^1^H NMR (400 MHz, DMSO-d_6_) δ = 13.44 (bs, 1H), 8.23-8.28 (m, 2H), 8.07 (s, 1H), 7.77 (s, 1H), 7.67-7.70 (m, 2H), 3.98 (s, 3H). LCMS: Calculated for C_16_H_10_F_3_N_3_O_4_S_2_ = 429.39, Observed = 430.0. HPLC = 98.158% (HPLC Column: Phenomenex Gemini-NX C18 (150 × 4.6 mm) 5micron, Mobile Phase A: 10 mM Ammonium acetate in water, Mobile Phase B: Acetonitrile).

### Compound 24: N-(4-(3, 5-difluorophenyl)thiazol-2-yl)-5-nitrothiophene-2-carboxamide (CAS: 2147485-29-6)

**Compound 24** was synthesized using scheme and procedure analogous to **compound 7** from commercially available 4-(3,5-difluoro-phenyl)-thiazol-2-ylamine (**CAS: 676348-23-5**, Fluorochem) and 5-Nitro-2-thiophencarboxylic acid.

1H NMR (400 MHz, DMSO-d6) δ = 13.39 (bs, 1H), 8.25-8.23 (m, 2H,), 8.01 (s, 1H), 7.67-7.65 (m, 1H), 7.23-7.21 (m, 1H). LCMS: Calculated for C_14_H_7_F_2_N_3_O_3_S_2_, = 367.34, Observed = 368.0, HPLC = 99.05% (HPLC Column: Phenomenex Gemini-NX C18 (150 × 4.6 mm) 5micron, Mobile Phase A: 10 mM Ammonium acetate in water, Mobile Phase B: Acetonitrile.

## Electronic supplementary material

Supplemtary Information

Supporting File SI2

Movie S1
