# Supplementary material for: Nitrothiophene carboxamides, a novel narrow spectrum antibacterial series: Mechanism of action and Efficacy
Source: Sci Rep. 2018 May 8;8:7263. doi: 10.1038/s41598-018-25407-7 (PMC5940854; doi:10.1038/s41598-018-25407-7)
Supplement: Supplementary file 1 — Supplemtary Information [file 41598_2018_25407_MOESM1_ESM.docx]

**Nitrothiophene carboxamides, a novel narrow spectrum antibacterial series: Mechanism of action and Efficacy**

Shahul Hameed P^1^, Nagakumar Bharatham^2^, Nainesh Katagihallimath^1^, Sreevalli Sharma^1^, Radha Nandishaiah^1^, Anirudh P Shanbhag^1^, Teby Thomas^3^, Riya Narjari^1^, Maitrayee Sarma^1^, Purnendu Bhowmik^1^, Prakruthi Amar^1^, Rajani Ravishankar^1^, Ramesh Jayaraman^4^, Kubendran Muthan^5^, Ramesh Subbiah^5^, Vasanthi Ramachandran^1^, V. Balasubramanian^1^ and Santanu Datta^1^*

^1^ BUGWORKS Research India Pvt. Ltd., Centre for Cellular & Molecular Platforms, National Centre for Biological Sciences, GKVK, Bellary Rd, Bengaluru, Karnataka 560065 India.

^2^ Centre for Cellular and Molecular Platforms (C-CAMP), National Centre for Biological Sciences, GKVK, Bellary Rd, Bengaluru, Karnataka 560065 India.

^3^ St. John’s Research Institute, 100 Feet Rd, John Nagar, Koramangala, Bengaluru, Karnataka 560034 India.

^4^ TheraIndx Lifesciences Pvt. Ltd., Sy No. 27, Deganahalli, Budihal Post, Nelamangala, Karnataka 562123 India.

^5^ Syngene International Ltd., Plot 2 & 3, Bommasandra Industrial Estate - Phase-IV, Bommasandra-Jigani Link Road, Bengaluru, Karnataka 560099 India.

| **compound ID** | **tolC** | **acrB** | **WT** | **acrD** | **acrF** | **acrZ** | **emrB** | **emrY** | **mdfA** | **bcr** | **mdtG** | **hsrA** | **mdtL** | **yciM** | **mdtK** | **emrE** | **macB** | **mdtB** |
| --- | --- | --- | --- | --- | --- | --- | --- | --- | --- | --- | --- | --- | --- | --- | --- | --- | --- | --- |
| **1 (2342425)** | 1.25 | 10 | >40 | >40 | >40 | >40 | >40 | >40 | >40 | >40 | >40 | >40 | >40 | >40 | >40 | >40 | >40 | >40 |
| **2 (2502792)** | 40 | 40 | >40 | >40 | >40 | >40 | >40 | >40 | >40 | >40 | >40 | >40 | >40 | >40 | >40 | >40 | >40 | >40 |
| **3 (2524542)** | 10 | 40 | >40 | >40 | >40 | >40 | >40 | >40 | >40 | >40 | >40 | >40 | >40 | >40 | >40 | >40 | >40 | >40 |
| **4 (1534259)** | 5 | 40 | >40 | >40 | >40 | >40 | >40 | >40 | >40 | >40 | >40 | >40 | >40 | >40 | >40 | >40 | >40 | >40 |
| **5 (4776326)** | 5 | 5 | >40 | >40 | >40 | >40 | >40 | >40 | >40 | >40 | >40 | >40 | >40 | >40 | >40 | >40 | >40 | >40 |
| **6 (5570930)** | 10 | 40 | >40 | >40 | >40 | >40 | >40 | >40 | >40 | >40 | >40 | >40 | >40 | >40 | >40 | >40 | >40 | >40 |
| **7 (3643444)** | 1.25 | 1.25 | >40 | >40 | >40 | >40 | >40 | >40 | >40 | >40 | >40 | >40 | >40 | >40 | >40 | >40 | >40 | >40 |
| **tetracycline** | 0.3 | 0.3 | 1.2 | 1.2 | 1.2 | 1.2 | 1.2 | 1.2 | 1.2 | 1.2 | 1.2 | 1.2 | 1.2 | 1.2 | 1.2 | 1.2 | 1.2 | 1.2 |

**Table S1:** MIC (µg/ml**)** values against a panel of *E. coli* efflux pump knockout strains. Emolecules vendor IDs were provided in parenthesis.

|  | **T_efflux50_, ∆Tm and MIC** | | | | | |
| --- | --- | --- | --- | --- | --- | --- |
| **compound** | **NIL** | **PAβN** | **7** | **12** | **15** | **20** |
| **1µM** | 51.98 | 200 | 88.05 | 66.41 | 61.52 | 69.24 |
| **9µM** | 51.98 | 200 | 200 | 67.52 | 72.51 | 71.14 |
| **27µM** | 51.98 | 200 | 200 | 67.98 | 81.24 | 72.65 |
| **81µM** | 51.98 | 200 | 200 | 68.06 | 124.25 | 104.21 |
| **100µM** | 51.98 | 200 | 200 | 68.17 | 200 | 200 |
| **200µM** | 51.98 | 200 | 200 | 68.34 | 200 | 200 |
| **400µM** | 51.98 | 200 | 200 | 68.25 | 200 | 200 |
| **∆Tm in ^ο^C** | NA | 3.84 | 4.13 | 0.68 | 1.75 | 1.86 |
| **WT MIC (µg/ml)** | NA | NA | >40 | 0.6 | 1.25 | 0.6 |

**Table S2:**  The rate of efflux is measured by T_efflux50_, it is the time taken for 50% of Nile red to be effluxed out in presence of the compounds. T_efflux50_ values estimated in Nile red competitive assay for NTC derivatives, PAβN and without any compound (NIL). ∆Tm estimated by thermal shift assay and MIC values on *E. coli* wild type strain also included for comparison. NA.; not applicable.

**
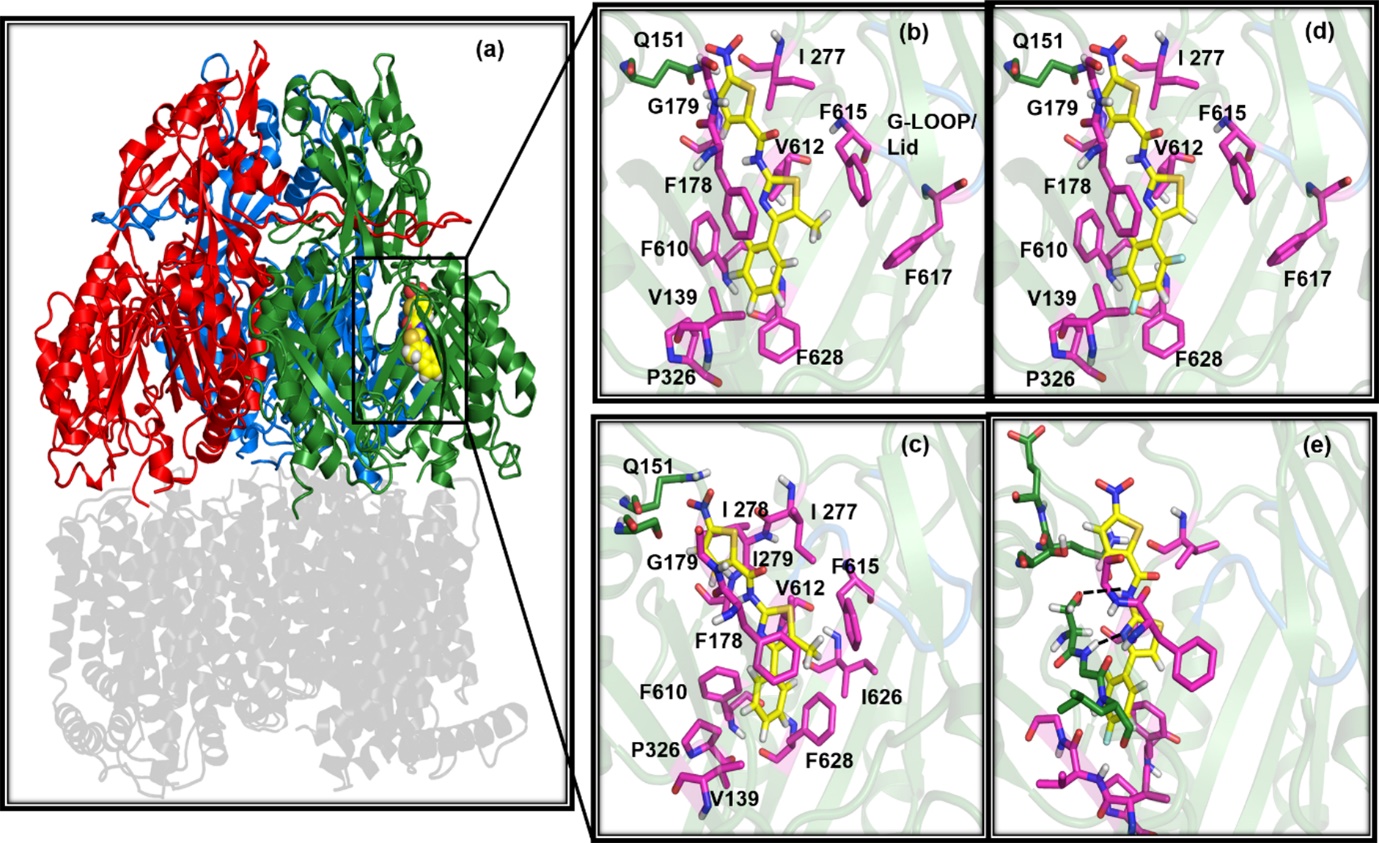
**

**Figure S1: AcrB binding mode prediction and evaluation:** (a) reduced model of AcrB used for MD simulation study. The three monomers of AcrB pump shown with different colours. One of the NTC derivative shown as yellow spheres in the hydrophobic pocket of binding/tight monomer. Inner membrane interacting portion of AcrB is omitted to generate the reduced model is shown in grey. The initial molecular docking predicted binding mode of (b) compound 7, (d) compound 12 and final snapshot of MD simulation from (c) compound 7 and (e) compound 12. Important hydrophobic contact residues are highlighted as magenta sticks and hydrophilic residues shown as green sticks. Hydrogen bond contacts shown as broken lines.


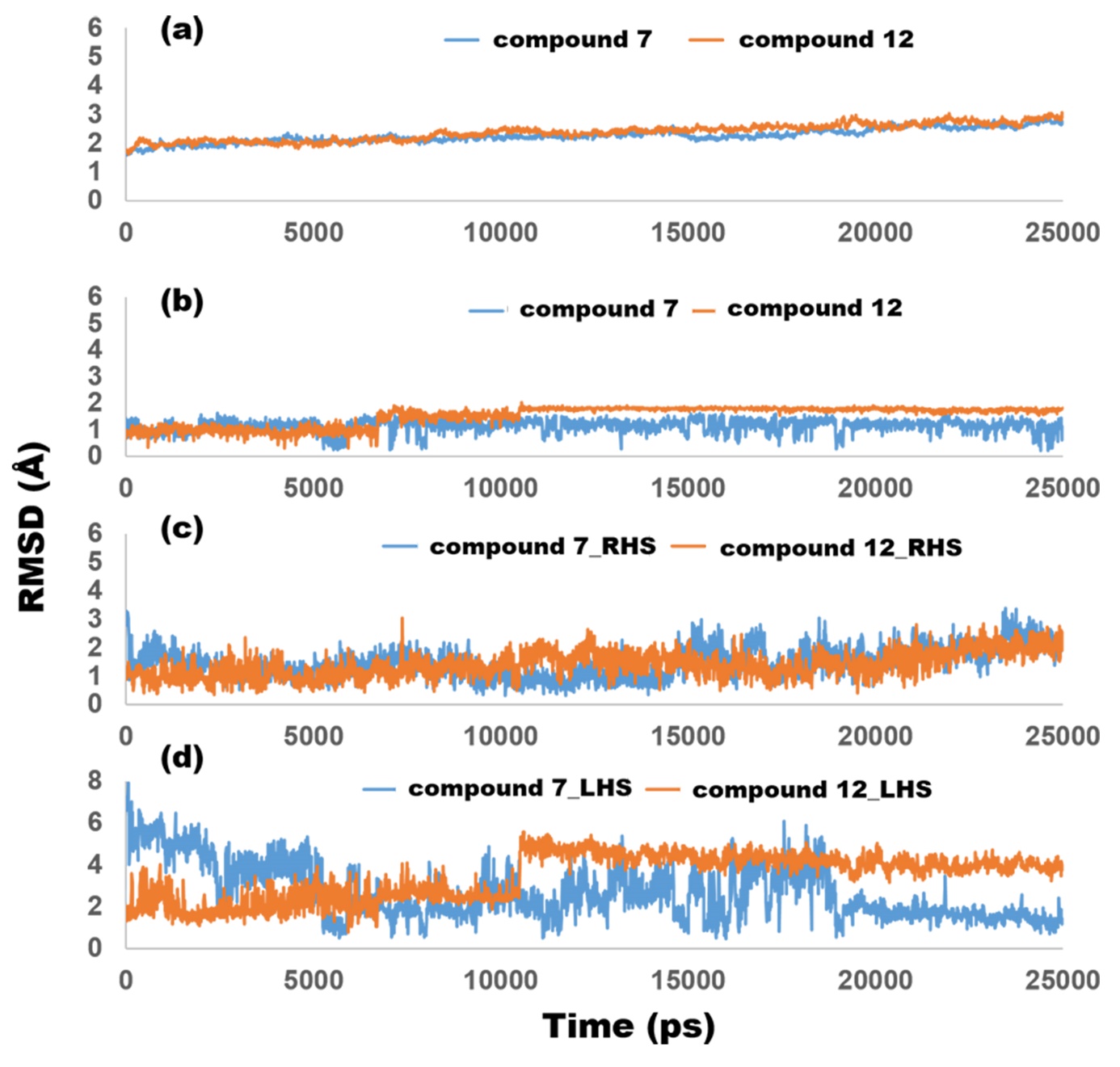


**Figure S2: RMSD analysed of MD trajectories.** (a) The backbone RMSD for reduced model of AcrB with compound 7 and compound 12, (b) RMS deviation for ligand atoms comparing with minimized binding orientation, (c) RMS deviation for ligand atoms of RHS ring comparing with minimized binding orientation (d) RMS deviation for ligand atoms of LHS phenyl ring comparing with minimized binding orientation.

| **Key Residues** | **compound 7** | **compound 12** |
| --- | --- | --- |
| V139 | -0.44 | -0.42 |
| V140 | -0.06 | -0.79 |
| G141 | -0.03 | -0.33 |
| Q151 | -0.92 | -0.68 |
| E152 | -0.29 | -0.68 |
| S155 | -0.84 | -1.24 |
| F178 | -2.58 | -2.19 |
| G179 | -0.79 | -0.92 |
| I277 | -1.68 | -1.14 |
| I278 | -1.28 | -0.3 |
| A279 | -1.18 | -1.21 |
| S287 | -0.86 | -2.84 |
| G288 | -0.28 | -1.36 |
| L289 | -0.22 | -0.74 |
| P326 | -0.56 | -0.37 |
| F610 | -1.05 | -1.16 |
| V612 | -1.49 | -0.54 |
| F615 | -0.86 | -0.21 |
| I626 | -0.34 | -0.05 |
| F628 | -0.66 | -0.04 |

**Table S3**: Residue wise energies calculated from AcrB:ligand complex trajectories by MM/GBSA method. Similar contributions are highlighted with green boxes and major differences in residue contributions are highlighted with yellow boxes. 0.3 considered as cut-off value for differentiation.

**
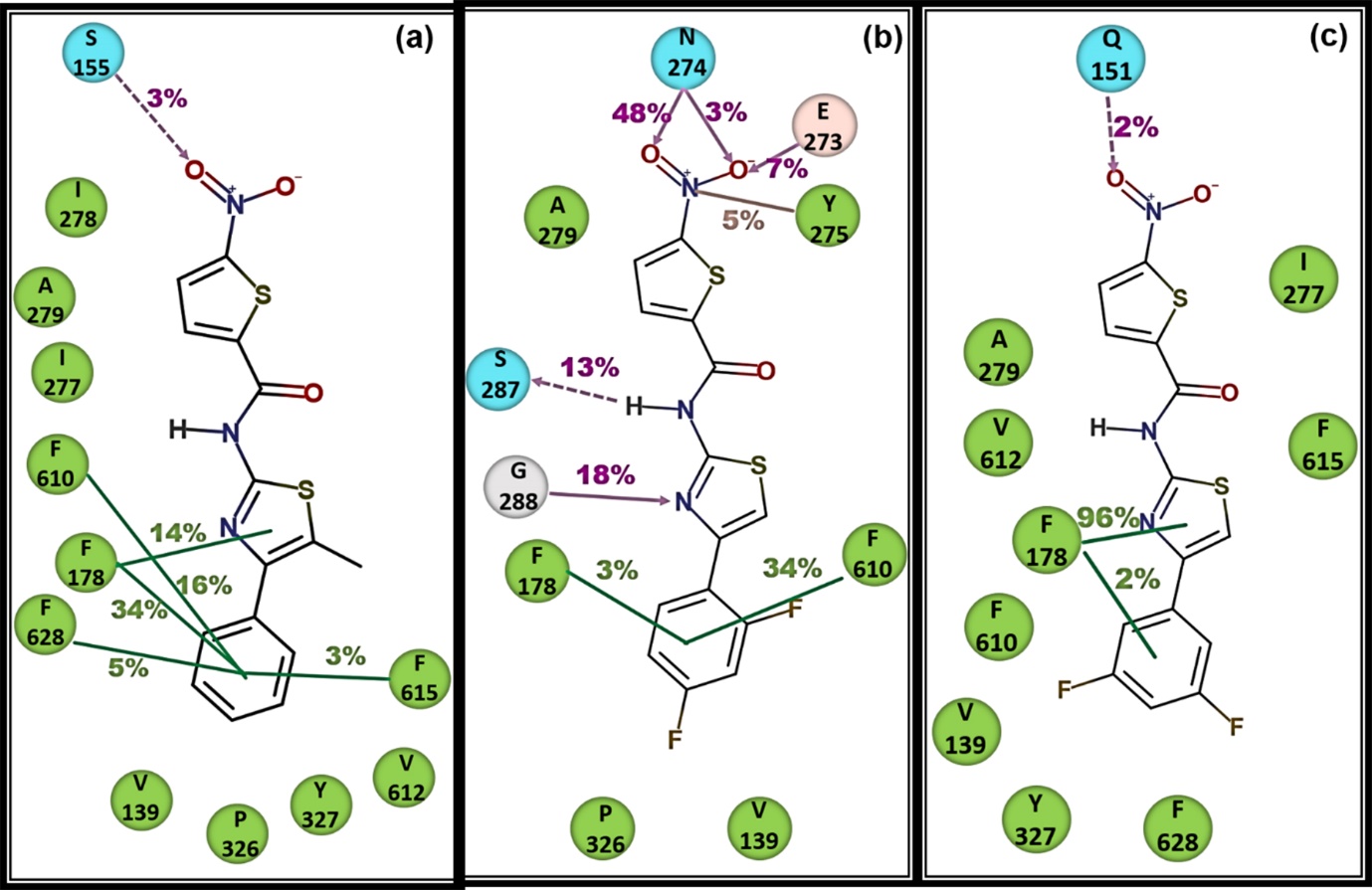
**

**Figure S3: Protein-ligand interaction fingerprints.** Protein-ligand interaction diagrams generated from MD simulation trajectories for (a) compound 7 (b) compound 12 and (c) compound 24. Hydrophobic contact points shown as green circles, hydrophilic contacts in cyan whereas negatively charged in pink and glycine in grey circles. Stacking interactions highlighted with green color lines and occupancy rates provided. Side chain hydrogen bond interactions highlighted with broken arrows whereas main chain interactions with solid arrows.

|  | **Conc. (μg/ml)** | | |
| --- | --- | --- | --- |
| **compound ID** | Range | MIC_50_ | MIC_90_ |
| **12** | 0.06 - 40 | 2.5 | 10 |
| **15** | 0.6 - 40 | 1.25 | 10 |
| **20** | 0.6 - 10 | 5 | 10 |
| **ciprofloxacin** | 0.015 - 4 | 4 | 4 |
| **meropenem** | 0.03 - 8 | 0.06 | 8 |
| **gentamicin** | 0.25 - >64 | 16 | >64 |
| **nitrofurantoin** | 5 - 160 | 20 | 80 |

**Table S4: MIC evaluation on clinical strains**. MIC_50_ (50% clinical isolates) and MIC_90_ (90% clinical isolates) estimated based on 75 *E.coli* clinical isolates MIC data.

|  | **Fold MIC (2x-16x) in agar plates** | **No. of colonies retrieved/ 10^9^ CFU/mL** | **Resistance mutation frequency** |
| --- | --- | --- | --- |
| **compound 11** |  |  |  |
| **3*10^9^ CFU/mL plated on** | 2x | 206 | 1.03E-07 |
|  | 4x | 58 | 2.90E-08 |
|  | 8x | 85 | 4.25E-08 |
|  | 16x | 16 | 8.00E-09 |
| **compound 15** |  |  |  |
| **2*10^9^ CFU/mL plated on** | 2x | 127 | 6.35E-08 |
|  | 4x | 79 | 3.95E-08 |
|  | 8x | 48 | 2.40E-08 |
|  | 16x | 11 | 5.50E-09 |
| **Mutations identified by sequencing *nfsB*: V143F, deletion at bp 34, insertion at bp 423 , substitution at bp 138** | | | |

**Table S5: Determination of mutation frequency**


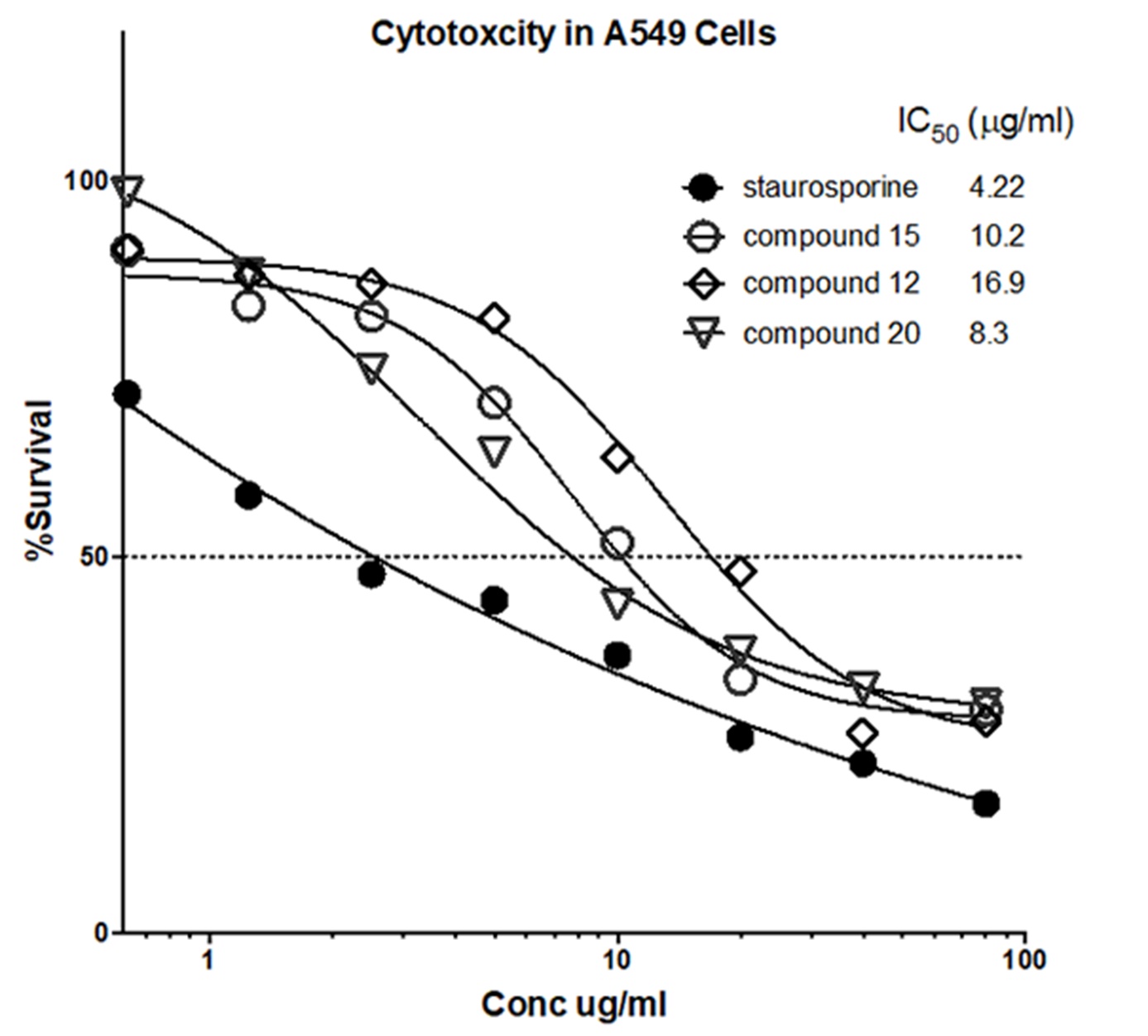


**Figure S4:** Cytotoxicity evaluation for compounds 12, 15 and 20 in A549 cells by MTS assay.


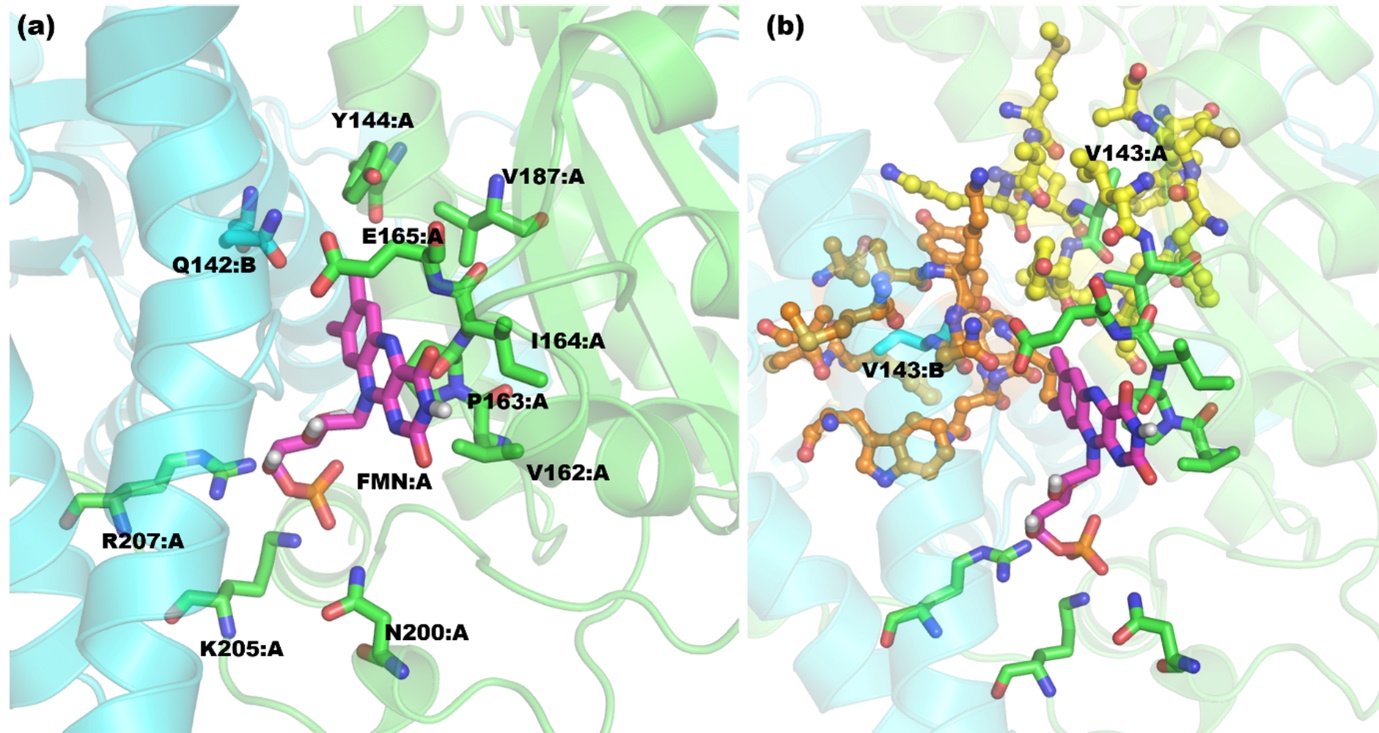


**Figure S5: NfsB Mutation mapping.** (a) FMN binding pocket (PDB ID: 1YKI) where most of the frame shift mutations observed in presence of compound 11 and compound 15. FMN co-factor highlighted with magenta sticks and NfsB residues highlighted with green sticks (monomer A) cyan sticks (monomer B). Protein represented as cartoon. (b) Point mutation residue V143 that is close to FMN pocket. Residues around 4Å of V143 highlighted as orange (chain A) ball and sticks and yellow (chain B) ball and sticks.

|  | **MIC (µg/ml)** | | | |
| --- | --- | --- | --- | --- |
| **compound ID** | **BW25113 (WT)** | **∆*nfsB*** | **∆*nfsA*** | **∆*nfsA* + ∆*nfsB*** |
| **7** | >40 | >40 | >40 | >40 |
| **12** | 0.6 | 2.5 | 0.6 | >40 |
| **14** | >40 | >40 | >40 | >40 |
| **15** | 1.25 | 5 | 0.6 | >40 |
| **20** | 1.25 | 2.5 | 0.6 | >40 |
| **nitrofurantoin** | 2.5 | 2.5 | 5 | 20 |

**Table S6: Evaluation of role of nitroreductases in NTC anti-bacterial activity.** MIC values were determined for a set of NTC derivatives and reference compound nitrofurantoin in *nfsA*, *nfsB* single and double knockouts.

| **Rates of compound conversion by Nitroreductases** | | |
| --- | --- | --- |
| **compound ID** | **NfsA activity (µM/min)** | **NfsB activity (µM/min)** |
| **nitrofurantoin** | 3.58 | 3.50 |
| **compound 7** | 0.64 | 6.17 |
| **compound 15** | 2.05 | 7.64 |
| **compound 12** | 2.30 | 6.26 |
| **compound 20** | 0.61 | 3.50 |
| **compound 14** | 0.20 | 1.31 |

**Table S7:** Rate of compound conversion, the table provides a comparison of the slopes of the initial velocities of the reactions in **Figure 5**.

| **Parameter** | **compound 12** | **compound 15** |
| --- | --- | --- |
| **Dose** | **2 mg/kg** | **2 mg/kg** |
| **K_el(β)_ (^-^h)** | 1.18 | 1.51 |
| **C_last_(µg/ml)** | 0.003 | 0.012 |
| **AUC_0-t_ (µg.h/ml)** | 0.57 | 0.9 |
| **AUC_t-∞_ (µg.h/ml)** | 0.003 | 0.008 |
| **AUC_0-∞_ (µg.h/ml)** | 0.57 | 0.911 |
| **extr AUC (%)** | 0.4 | 0.9 |
| **AUMC_0-∞_** | 0.26 | 0.17 |
| **t_1/2_ (h)** | 0.6 | 0.5 |
| **CL (L/h/kg)** | 3.52 | 2.20 |
| **V_ss_ (L/kg)** | 1.64 | 0.40 |

**Table S8:** NCA PK parameters of compound 12 & 15 in mice following a single IV dose

| **Parameter** | **compound 12** | | **compound 15** | |
| --- | --- | --- | --- | --- |
| **Dose** | **10 mg/kg** | | **10 mg/kg** | **100 mg/kg** |
| C_max_(µg/ml) | 0.65 | | 1.2 | 3.8 |
| T_max_(h) | 0.25 | | 0.25 | 0.25 |
| AUC_(0-t)_ (µg.h/ml) | 0.75 | | 0.75 | 10.3 |
| AUC_(t-∞)_ (µg.h/ml) | 0.004 | | 0.01 | NE |
| AUC_(0-∞)_ (µg.h/ml) | 0.76 | | 0.76 | NE |
| ext AUC (%) | 0.5 | | 1.5 | NE |
| Ke (h) | 0.67 | | 0.14 | NE |
| t_1/2_(h) | 1.03 | | 4.82 | NE |
| **Estimation of oral Bioavailability** | | | | |
| AUC (IV, 2 mg/kg) | 0.57 | 0.91 | | 0.91 |
| AUC (PO, 10mg/kg) | 0.76 | 0.75 | | 10.3 |
| **F (%)** | 26.7 | 16.5 | | 22.6 |

**Table S9:** NCA PK parameters of compound 12 & 15 in mice following a single oral dose.

NE: Not Estimated due to lack of declining linear terminal phase.

| **Study No.** | **Test Strain** | **Comparison vs. Untreated Controls** | **Log_10_CFU Mean Diff.** | **P Value** |
| --- | --- | --- | --- | --- |
| **TI-2014-007** | ∆*acrB* JW0451-2 | ciprofloxacin 100mg/kg | 3.477 | 0.0001 |
| **TI-2014-007** | ∆*acrB* JW0451-2 | compound 15 10mg/kg | 0.2933 | 0.8274 |
| **TI-2014-007** | ∆*acrB* JW0451-2 | compound 15 30mg/kg | 1.943 | 0.0003 |
| **TI-2014-007** | ∆*acrB* JW0451-2 | compound 15 100mg/kg | 2.436 | 0.0001 |
| **TI-2014-008** | BW25113 | ciprofloxacin 100mg/kg | 3.212 | 0.0001 |
| **TI-2014-008** | BW25113 | compound 15 30mg/kg | 0.9167 | 0.0094 |
| **TI-2014-008** | BW25113 | compound 15 100mg/kg | 1.643 | 0.0002 |
| **TI-2014-008** | ∆*acrB* JW0451-2 | ciprofloxacin 100mg/kg | 3.098 | 0.0001 |
| **TI-2014-008** | ∆*acrB* JW0451-2 | compound 15 30mg/kg | 1.497 | 0.0001 |
| **TI-2014-008** | ∆*acrB* JW0451-2 | compound 15 100mg/kg | 2.253 | 0.0002 |
| **TI-2014-023** | Clinical Isolate N. 371 | ciprofloxacin 100mg/kg | 3.103 | 0.0001 |
| **TI-2014-023** | Clinical Isolate N. 371 | compound 15 100mg/kg | 1.553 | 0.0003 |
| **TI-2014-023** | Clinical Isolate N. 371 | compound 12 100 mg/kg | 1.907 | 0.0001 |

**Table S10:** Dunnett's multiple comparisons test following per oral treatment in a neutropenic thigh infection model using GraphPad Prism v7.0 for Mac OS. P-value < 0.05 is significant.

**Figure S6:** Coomassie stained SDS-PAGE of affinity purified NfsA and NfsB protein: Lanes‑1 NfsA, 2‑NfsB and 3‑Molecular weight marker.


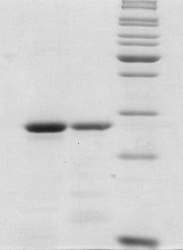


**1 2 3 KDa**

200

150

100

85

70

60

50

40

30

20

10


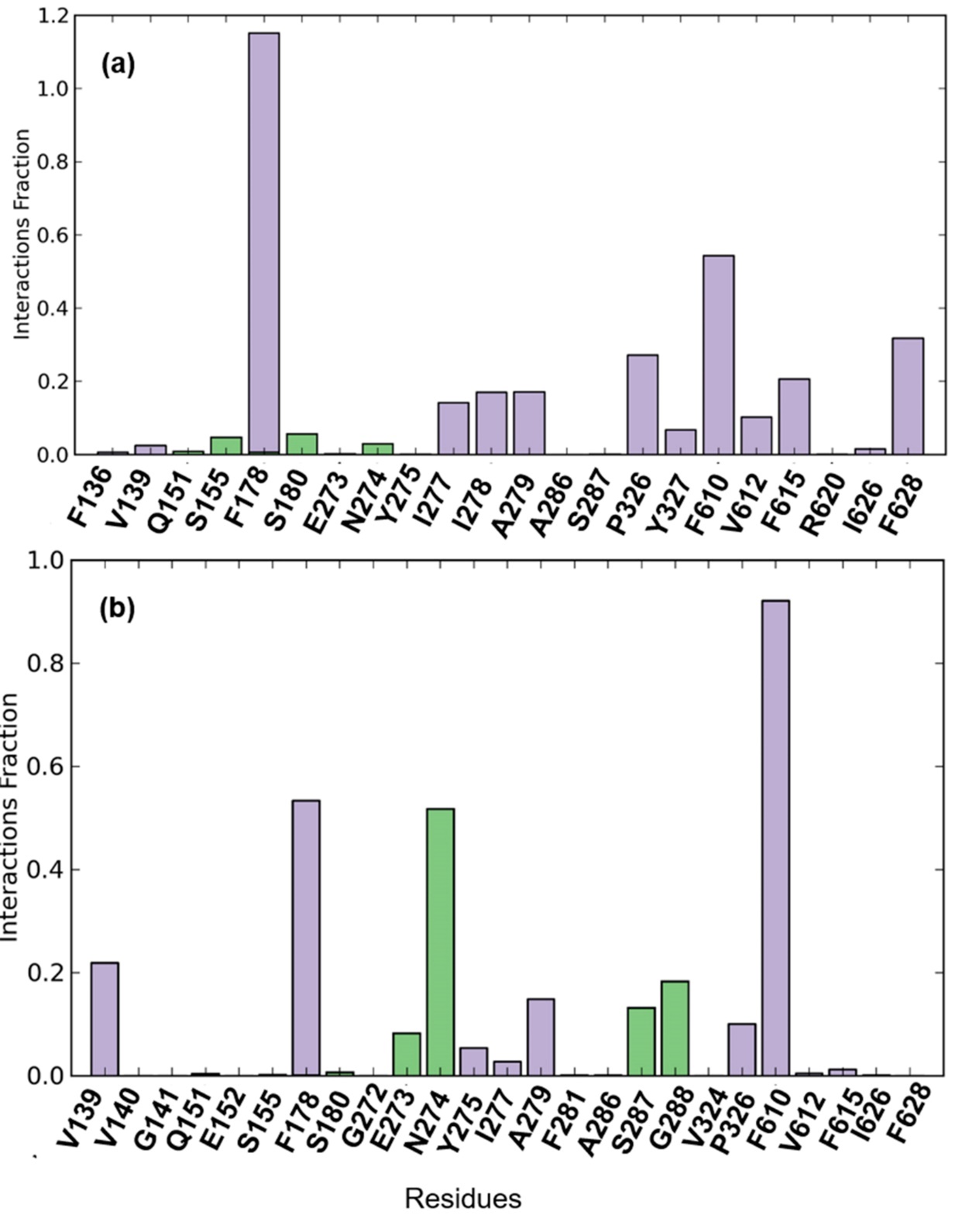


**Figure S7: % occupancy of interactions with key AcrB residues.** Interaction pattern of (a) compound 7 and (b) compound 12 with AcrB binding pocket residues


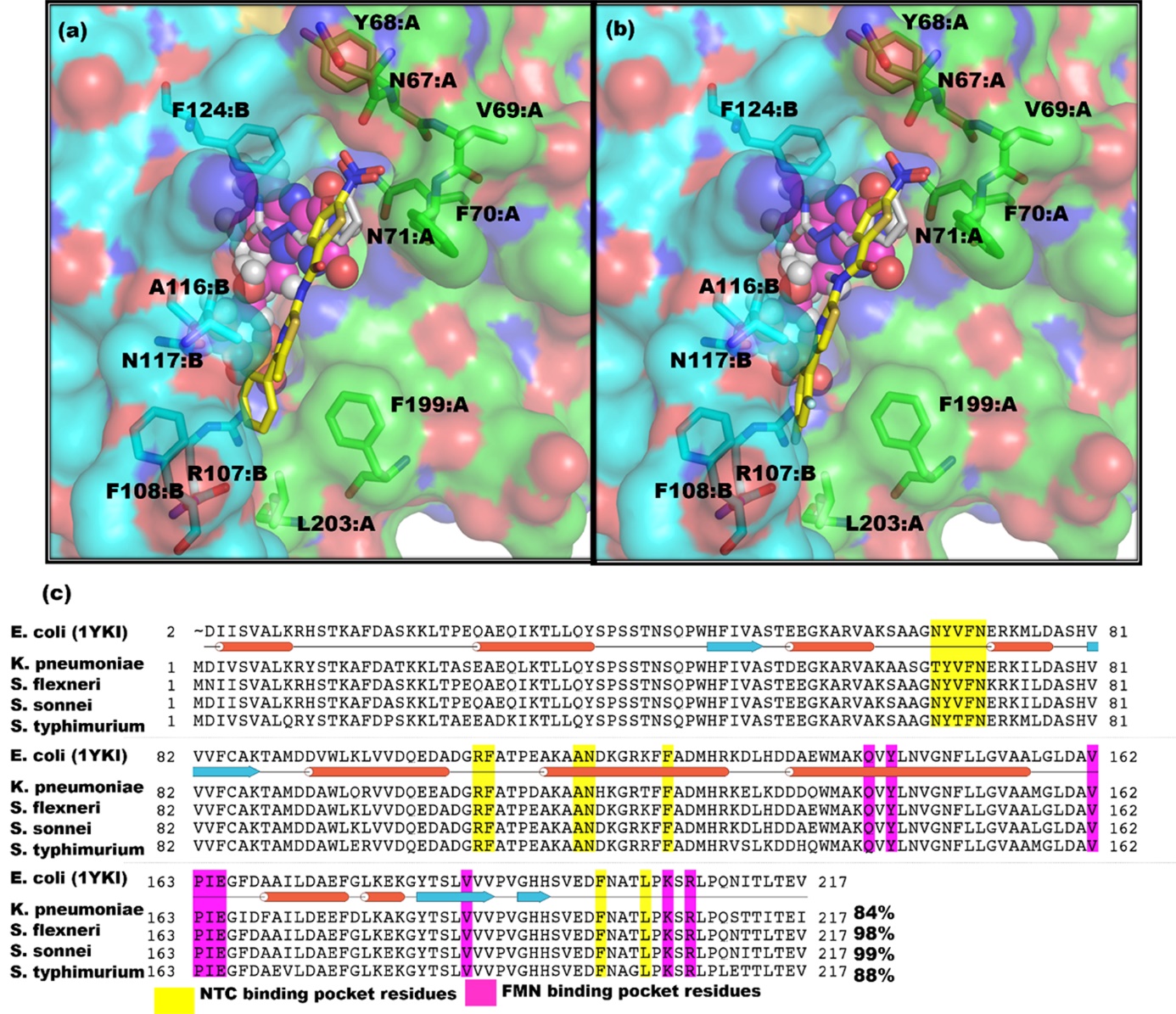

**Figure S8: NTC derivatives binding mode with NfsB protein.** (a) The compound 7 and (b) compound 12 molecular docking predicted binding modes with NfsB protein. FMN shown as magenta spheres whereas NTC derivatives shown as yellow sticks. NfsB protein dimer shown as green (chain A) and cyan (chain B) colour surface and active site residues highlighted as sticks and labelled accordingly. The nitrofurazone of co-crystal structure shown as white sticks. (c) Sequence alignment of E. coli NfsB protein sequence with other probable identical proteins in various bacterial pathogens.

| **compounds** | **WT** | **∆*acrB*** | **∆*nfsAB* & ∆*acrB*+nfnB** | ***Klebsiella pneumoniae*** | ***Shigella flexneri*** | ***Shigella sonnei*** | ***Salmonella typhimurium*** |
| --- | --- | --- | --- | --- | --- | --- | --- |
| **12** | 0.6 | 0.3 | 0.3 | 2.5 | 10 | 5 | 2.5 |
| **15** | 1.25 | 0.6 | 0.6 | 5 | ND | ND | ND |
| **20** | 1.25 | 0.3 | 0.6 | 10 | ND | ND | 10 |

**Table S11: MIC determination on other pathogens.** MICs were determined on triple knockout (**∆*nfsAB*&∆*acrB***) with *nfnB* complementation as well as *Klebsiella* *pneumoniae* ATCC13883. Clinical strains of *Shigella flexneri*, *Shigella sonnei* and *Salmonella typhimurium* also utilized for MIC studies.

**Figure S9:** Coomassie stained SDS-PAGE depicting the isolation of AcrB labelled as following lane 1: Lysate 2: Lysate after removal of cell debris 3: Soluble fraction 4: Membrane fraction 5: Flowthrough 6: Wash fraction 7: AcrB protein (affinity purified) 8: High range marker (Biorad)


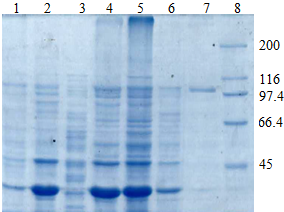


KDa


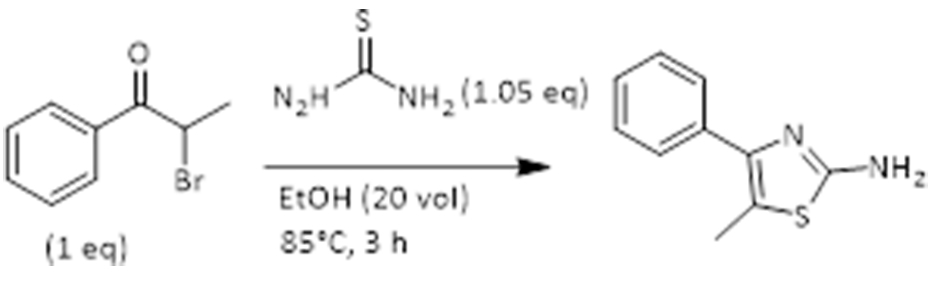


**Figure S10:** Synthesis of 5-methyl-4-phenylthiazol-2-amine (7a)


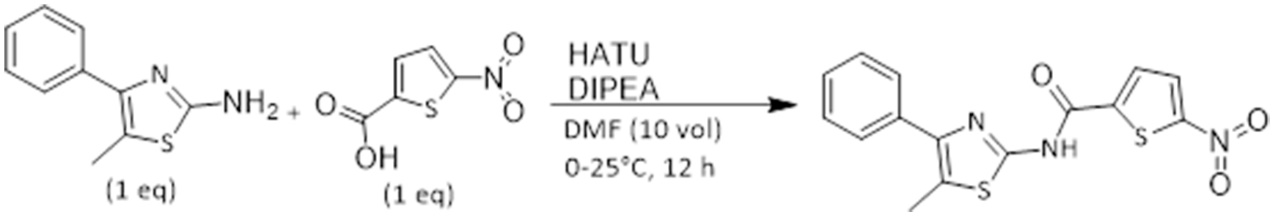


**Figure S11**: Synthesis of N-(5-methyl-4-phenylthiazol-2-yl)-5-nitrothiophene-2-carboxamide


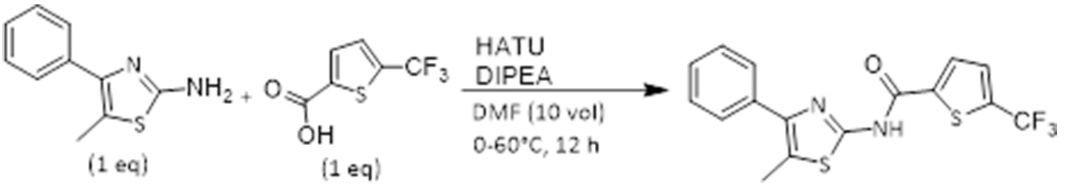


**Figure S12:** Synthesis of N-(5-Methyl-4-phenylthiazol-2-yl)-5-(trifluoromethyl) thiophene-2-carboxamide


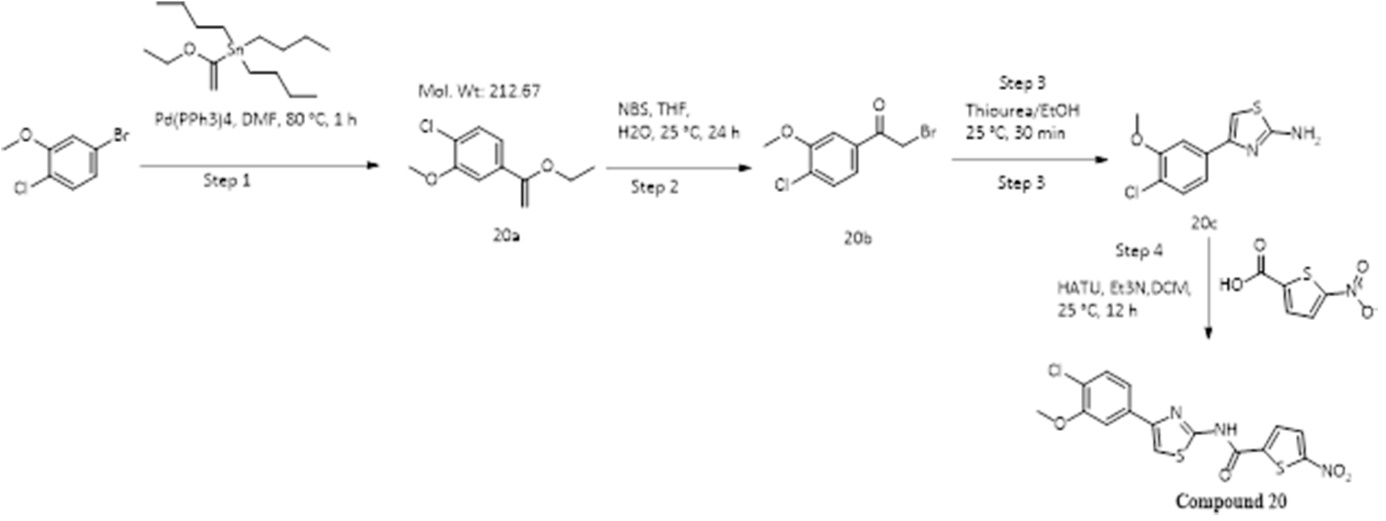


**Figure S13:** Synthesis of N-(4-(4-chloro-3-methoxyphenyl)thiazol-2-yl)-5-nitrothiophene-2-carboxamide

**
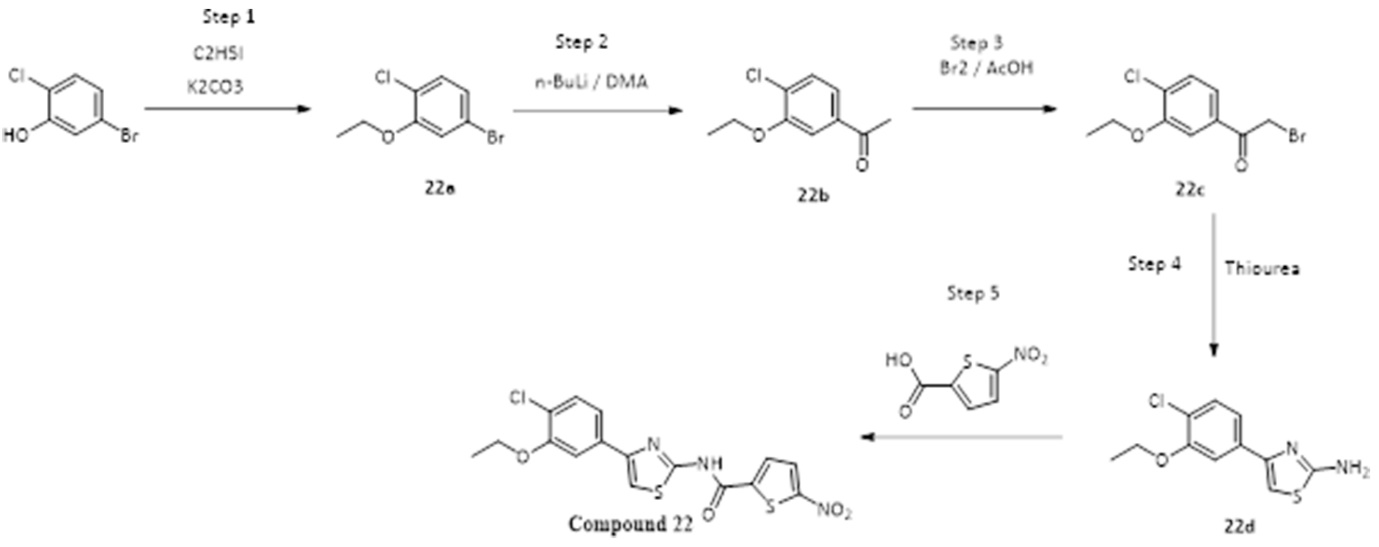
**

**Figure S14:** Synthesis of N-(4-(4-chloro-3-ethoxyphenyl)thiazol-2-yl)-5-nitrothiophene-2-carboxamide


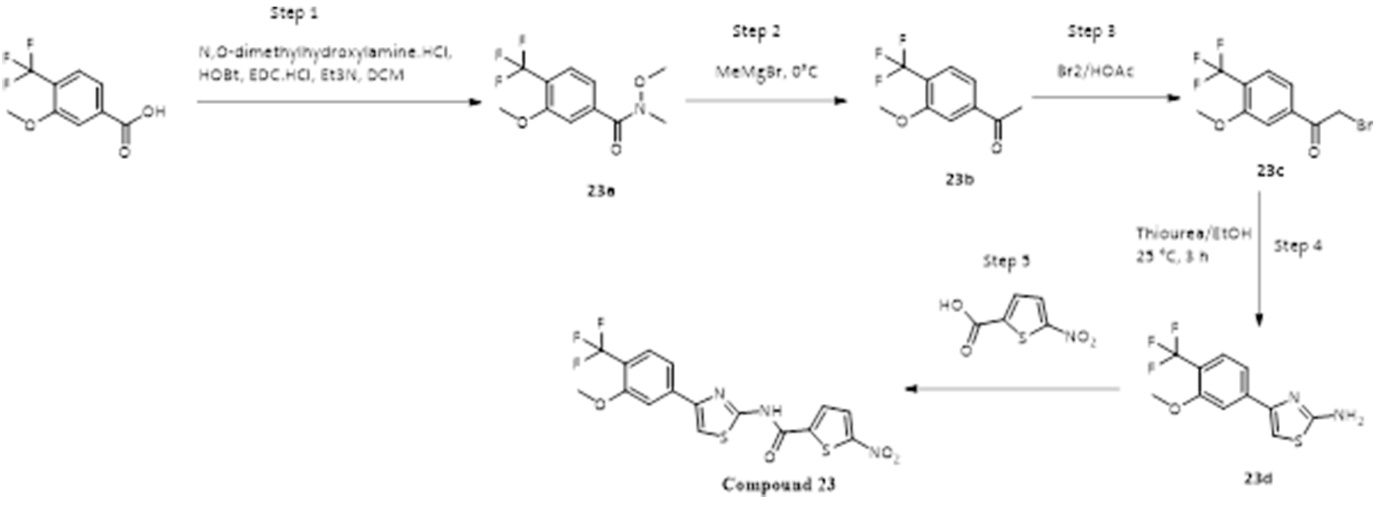


**Figure S15:** Synthesis of N-(4-(3-methoxy-4-(trifluoromethyl)phenyl)thiazol-2-yl)-5-nitrothiophene-2-carboxamide
